# ImmTACs overcome cytotoxic T cell suppression

**DOI:** 10.1093/immadv/ltag020

**Published:** 2026-08-14

**Authors:** Lan Huynh, Abdullah Aljohani, Amal Alsubaiti, Tressan Grant, Alexandra Chapman, Gwilym Phillips, Jonathan Chamberlain, Alice Hayward-Wills, Ute Jungwirth, Mariolina Salio, Christopher J Holland, Christoph Wülfing

**Affiliations:** School of Cellular and Molecular Medicine, University of Bristol, Bristol, United Kingdom; School of Cellular and Molecular Medicine, University of Bristol, Bristol, United Kingdom; School of Cellular and Molecular Medicine, University of Bristol, Bristol, United Kingdom; School of Cellular and Molecular Medicine, University of Bristol, Bristol, United Kingdom; Department of Life Sciences, University of Bath, Bath, United Kingdom; ImmunoCore Ltd., Abingdon, United Kingdom; ImmunoCore Ltd., Abingdon, United Kingdom; ImmunoCore Ltd., Abingdon, United Kingdom; School of Cellular and Molecular Medicine, University of Bristol, Bristol, United Kingdom; Newcastle Drug Discovery Group, Translational and Clinical Research Institute, Newcastle University, Newcastle upon Tyne, United Kingdom; ImmunoCore Ltd., Abingdon, United Kingdom; ImmunoCore Ltd., Abingdon, United Kingdom; School of Cellular and Molecular Medicine, University of Bristol, Bristol, United Kingdom

**Keywords:** cancer immunotherapy, T cell exhaustion, synthetic T cell receptor engager, actin dynamics

## Abstract

**Introduction:**

Synthetic T cell receptor (TCR) engagers are cancer therapeutics that activate T cells through recognition of antigens on the tumor cell surface. Immune-mobilizing monoclonal TCRs against cancer (ImmTAC) are composed of the extracellular domains of the alpha and beta chains of an affinity-enhanced TCR recognizing a tumor-associated antigenic peptide-major histocompatibility complex (MHC) complex linked to an scFv fragment of an antibody against CD3ε. A first-in-class ImmTAC, Tebentafusp, is approved for the treatment of metastatic uveal melanoma. Here we asked how ImmTACs activate human primary cytotoxic T lymphocytes (CTL) in response to antigen-presenting tumor target cells.

**Materials and methods:**

We used a recently established experimental strategy to generate active and matched suppressed CTL *in vitro*.

**Results:**

ImmTACs could elicit tumor cell cytolysis in response to endogenous antigen presentation by both active and suppressed CTL, but much less so for IFNγ secretion, in a manner dependent on the engager affinity for CD3ε. ImmTACs did so by enhancing the efficient execution of subcellular CTL polarization steps required for effective cytolysis and could trigger calcium signaling.

**Conclusion:**

These data establish that ImmTACs are powerful inducers of CTL cytotoxicity and do so with a comparable efficacy and mechanism as direct engagement of a TCR by peptide-MHC. ImmTACs retain this capability under suppressive conditions comparable to those in the tumor microenvironment.

## Graphical Abstract

**Graphical Abstract:**
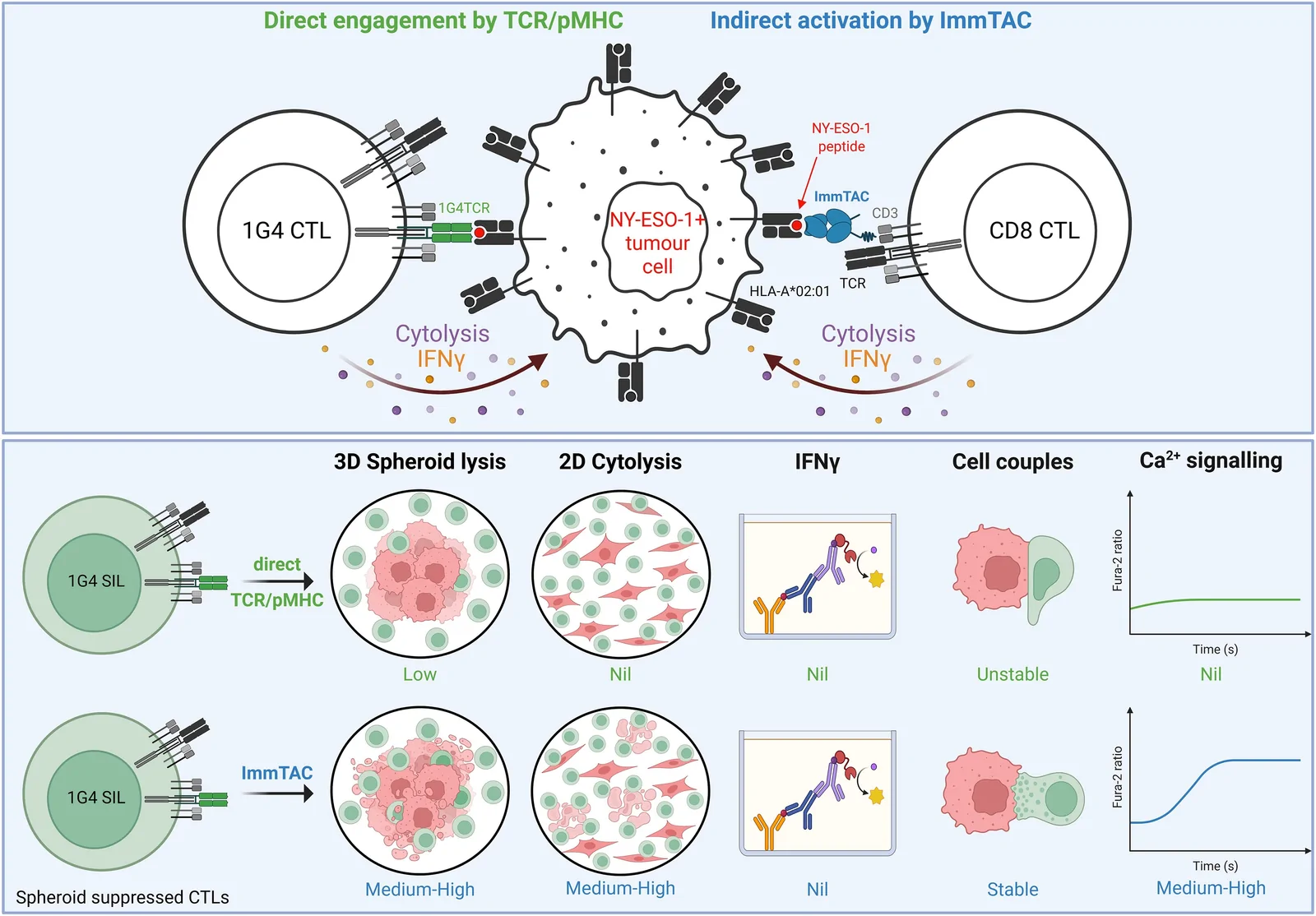

## Introduction

Cancer commonly triggers an antitumor immune response with the potential to cure the disease. Key immune effectors are cytotoxic T lymphocytes (CTL) that can directly kill tumor target cells [1]. To do so, the T cell receptor (TCR) of a CTL needs to recognize a peptide derived from a tumor-associated antigen or neoantigen as presented by major histocompatibility complex (MHC) molecules on the tumor cell surface. Common challenges to an effective T cell-mediated antitumor immune response are insufficient numbers of tumor-reactive T cells and a suppressive tumor microenvironment (TME) that impairs the function of tumor-infiltrating T cells [1]. Multiple therapeutic strategies aim to increase the number of T cells that can be activated in the TME. These include adoptive cell therapies with autologous *in vitro* expanded tumor-infiltrating lymphocytes and autologous T cells virally engineered to express a tumor-reactive TCR or a chimeric antigen receptor [2, 3]. Alternatively, synthetic T cell engagers aim to activate all T cells present in the TME [4]. As a substantial fraction of T cells in the TME are bystander T cells [5, 6], as they don't express a tumor antigen-reactive TCR, this strategy can substantially increase the number of activated T cells.

The initial design of bispecific T cell engagers connects a tumor-specific cell surface protein recognized by an antibody fragment to the CD3ε subunit of the TCR [4, 7]. However, with only 10% of the proteome consistently expressed on the cell surface across multiple cell types [8], the number of proteins that can be recognized by such engagers can be limiting. To access the intracellular proteome for tumor cell recognition, immune-mobilizing monoclonal TCRs against cancer (ImmTAC) recognize peptide-MHC complexes on the tumor cell surface. ImmTACs are bispecific T cell engagers composed of the disulfide-linked extracellular domains of the alpha and beta chains of an affinity-enhanced TCR recognizing a tumor-associated antigenic peptide presented by MHC, linked to an scFv fragment of an antibody against CD3ε [9]. Tebentafusp, an ImmTAC recognizing a peptide derived from gp100 as presented by HLA-A*02:01, is clinically approved for the treatment of metastatic uveal melanoma [10, 11]. Patient data show enhanced expression of molecules associated with T cell activation in tumor biopsies [12]. This is consistent with *in vitro* data showing induction of tumor cell killing and T cell IFNγ secretion by ImmTACs [13].

However, questions about the mechanism of action of ImmTACs remain unresolved. ImmTACs can activate CTL *in vitro* [14], but does this extend to suppressed CTL, mimicking CTL in the TME? Effective CTL-mediated target cell killing requires the sequential execution of a series of subcellular polarization steps to stabilize the CTL target cell couple [15–17]. Do ImmTACs regulate such subcellular polarization? Proximal T cell signaling, especially the elevation of the cytoplasmic calcium concentration, is associated with effective cytokine secretion in CTL [18]. Do ImmTACs regulate such signaling? Using three-dimensional tissue culture, we have recently established a strategy to *in vitro* generate active and matched suppressed CTL that closely resemble exhausted CTL in the TME [19]. We have employed this approach to address the above questions regarding the mechanism of action of ImmTAC molecules. Understanding this mechanism is important in the rational development of future therapies that combine ImmTACs with other immune therapies such as antibodies to block inhibitory receptors.

Understanding the mechanism of action of ImmTACs is also of interest in comparison to other therapies that redirect T cells toward tumor target cells. CAR T cells are effective in treating B cell-derived cancers but need to rely on T cell signal transduction that is inefficient in comparison to direct TCR engagement [20]. Is ImmTAC-mediated T cell activation in contrast as effective as that triggered by direct engagement of the TCR by peptide-MHC? BiTEs use a similar scFv fragment of an antibody against CD3ε as ImmTACs [7]. In addition, synthetic design approaches have recently been applied to generate new specific binders to peptide-MHC complexes, including binders to the same peptide-MHC complex investigated here [21–23]. In their current iteration, these binders have affinities for the peptide-MHC complexes in the low nM range, as opposed to the 18 pM *K*_D_ of the ImmTAC used here [9]. They can be incorporated into synthetic TCR engagers analogous to an ImmTAC and have been shown to trigger effective T cell activation in this form [21]. Investigating the mechanism of action of ImmTACs thus should inform comparable T cell engagers [24].

We use an ImmTAC recognizing the peptide 157–165 of the tumor-associated antigen NY-ESO-1 presented by HLA-A*02:01 [24] as a model for this class of therapeutics and compare its activity to direct activation of CTL lentivirally transduced to express the 1G4 TCR that recognizes the same peptide/MHC complex [25]. As effectors, we use active and *in vitro* suppressed CTL, generated following activation of primary human CTL with tumor cell spheroids [19]. As targets, we use the melanoma cell lines Mel624 and A375, both of which express low amounts of NY-ESO-1 [19]. We show that under suppressive conditions ImmTACs are effective at eliciting cytolysis but not IFNγ secretion, and this process is fine-tuned by anti-CD3ε affinity [13]. Using live cell imaging of the interaction of suppressed CTL with tumor target cells, we show that ImmTACs behave similarly to direct TCR engagement by peptide-MHC in enhancing the subcellar polarization of CTL and leading to elevation of the intracellular calcium concentration. Together, our data suggest that ImmTACs are effective reagents to trigger cytolysis even under suppressive conditions with a mechanism that closely resembles direct TCR engagement by peptide-MHC.

## Results

### Immune-mobilizing monoclonal T cell receptors against cancer effectively trigger cytolysis as a function of antigen dose and immune-mobilizing monoclonal T cell receptors against cancer affinity for CD3ε

**Figure 1:**
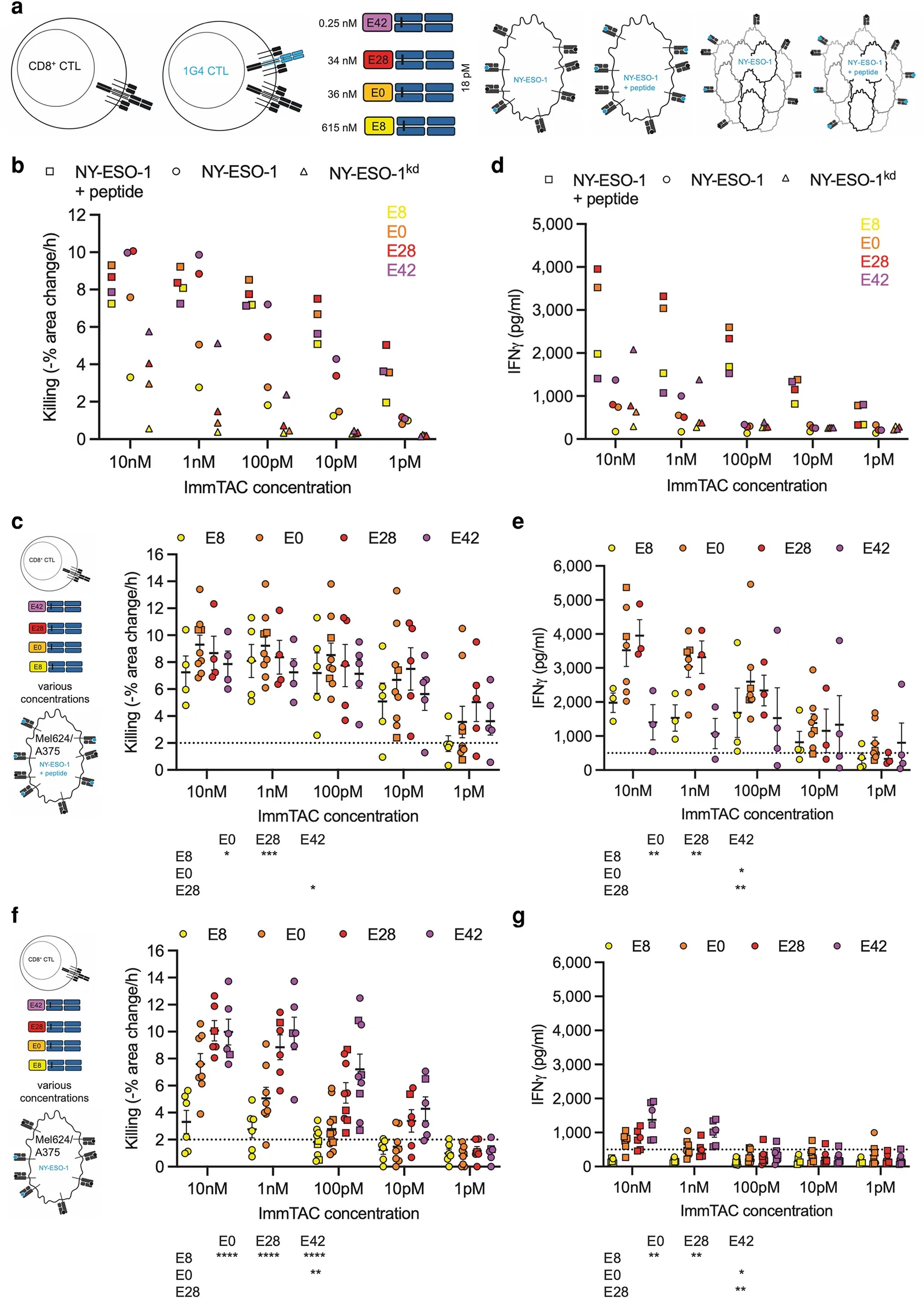
ImmTACs trigger CTL effector function as a function of antigen dose and ImmTAC affinity for CD3ε. **a** Graphical depiction of cell types and ImmTACs used: non-transduced CTL, CTL transduced to express the 1G4 TCR, ImmTACs with different anti-CD3ε scFv fragments, Mel624 or A375 melanoma cells with only endogenous NY-ESO-1 processing, Mel624 or A375 melanoma cells in the presence of 2 µg/ml NY-ESO-1_157–165_ peptide, and the corresponding Mel624 spheroids. Dot indicates the NY-ESO-1_157–165_ peptide. **b** Killing of Mel624 or A375 tumor target cells (incubated with 2 µg/ml NY-ESO-1 peptide) by non-transduced CTL in the presence of the indicated concentration of the given ImmTAC version; data pooled for Mel624 (circle) and A375 (square) tumor target cells; mean ± SEM. A broken line indicates the expected level of killing in the absence of antigen. 5–9 independent experiments. Statistical significance determined by paired mixed-effects analysis and given as main effects of ImmTAC version and concentration in the two tables at the right. **c** IFNγ amounts in supernatants of Mel624 (circle) and A375 (square) tumor target cells incubated with 2 µg/ml NY-ESO-1 peptide and interacting with non-transduced CTL in the presence of the indicated concentration of the given ImmTAC version; data pooled; mean ± SEM. A broken line indicates the expected level of IFNγ secretion in the absence of antigen. 3–8 independent experiments. Statistical significance determined by paired mixed-effects analysis and given as in (b). **d** Killing of Mel624 (circle) and A375 (square) tumor target cells by non-transduced CTL in the presence of the indicated concentration of the given ImmTAC version; mean ± SEM. 9–11 independent experiments, with not all ImmTAC concentrations present in all experiments. Statistical significance determined by mixed-effects analysis and given as in (b). **e** IFNγ amounts in supernatants of Mel624 (circle) and A375 (square) tumor target cells interacting with non-transduced CTL in the presence of the indicated concentration of the given ImmTAC version; mean ± SEM. 7–10 independent experiments, with not all ImmTAC concentrations present in all experiments. Statistical significance determined by paired mixed-effects analysis and given as in (b). *P < 0.05, **P < 0.01, ***P < 0.001, ****P < 0.0001.

As reference data for an investigation of ImmTACs in a suppressive environment, we first determined the ability of ImmTACs to activate CTL as a function of antigen dose, ImmTAC dose, and ImmTAC affinity for CD3ε. We used ImmTAC molecules that recognize the peptide 157–165 (SLLMWITQC) of the tumor-associated antigen NY-ESO-1 presented by HLA-A*02:01 [24] with a high affinity of *K*_D_ = 18 pM [9]. Based originally on the anti-CD3ε antibody UCTH1 [26], we used four ImmTACs differing in binding to CD3ε with affinity increasing from E8 < E0 = E28 < E42 (*K*_D_ = 615 nM, 36 nM, 34 nM, 0.25 nM) and with faster on and off rates of E28 than E0 (*t*_1/2_ = 4.6 min, 7.6 min) [13] (Fig. 1a). We used human *in vitro* primed CTL as previously characterized [19], *per se* (“CD8^+^ CTL”) or lentivirally transduced to express the 1G4 TCR that recognizes the same NY-ESO-1_157–165_/HLA-A*02:01 complex (“1G4 CTL”) [25] (Fig. 1a) to allow a comparison with CTL activation by direct engagement of the TCR by peptide-MHC. As tumor target cells, we predominantly used Mel624 melanoma cells, and in a few experiments, A375 melanoma cells. Both cell lines express small amounts of NY-ESO-1 that allow for limiting activation of 1G4 CTL [19]. To generate saturating amounts of antigen, we have incubated the cell lines with 2 µg/ml of exogenous NY-ESO-1_157–165_ peptide (Fig. 1a). Mel624 cells can effectively be grown as spheroids (Fig. 1a), and incubation of such spheroids with 1G4 CTL induces suppression comparable to CTL exhaustion in tumors [19].

In response to target cells incubated with exogenous NY-ESO-1 peptide as a maximal stimulus, all four ImmTACs triggered cytolysis (Fig. 1b), with only E8 being less effective, as determined in an imaging-based time-resolved overnight killing assay [16]. Cytolysis was saturated at an ImmTAC concentration of 100 pM (Supplementary Figure S1a), except for E28 at 10 pM. In patients, ImmTACs are given weekly, leading to fluctuating *in vivo* concentrations [24]. In a phase 1 clinical trial of a comparable NY-ESO-1-directed ImmTAC as used here, ImmTAC plasma concentrations reach 50 pM at the time of administration [24] with uncertain uptake into tumors. We, therefore, use 100 pM in single-concentration experiments later in this manuscript. IFNγ secretion, as determined in the supernatants at the end of the killing assay, was less effectively triggered and showed greater differences between the different ImmTAC versions. IFNγ secretion was most efficiently triggered by the E0 and E28 ImmTACs, requiring between 100 pM and 1 nM for saturation (Fig. 1c, Supplementary Figure S1a). Less effective induction of IFNγ secretion in comparison to cytolysis is similarly found in the direct activation of 1G4 CTL by the same melanoma cells [19]. A bell-shaped curve linking ImmTAC affinity for CD3ε to cytokine secretion has been previously described [13, 27], consistent with the results of 100 pM, 1 nM, and 10 nM ImmTAC (Fig. 1d, e, Supplementary Figure S1a). Substantial variability between experimental repeats is a documented feature of using primary human CTL [19] and is accounted for by executing multiple repeats.

CTL activation by ImmTACs in response to endogenous antigen presentation was substantially less effective. The E8 ImmTAC could no longer trigger effective cytolysis across the entire ImmTAC dose range (Fig. 1f, Supplementary Figure S1a). E28 and E42 were most effective but now required a concentration of 1 nM for saturating cytolysis, an approximate 10- to 100-fold shift in the dose–response curve compared to the cytolysis in the presence of saturating amounts of peptide (Fig. 1c). Such cytolysis was still antigen-dependent, as shown with NY-ESO-1 knockdown in Mel624 cells [19] (Supplementary Figure S1b, c). Killing only occurred at a reduced level and at ImmTAC concentrations above 100 pM for E42 ImmTAC and at 10 nM for E28 and E0 molecules (Supplementary Figure S1b). In contrast to cytolysis, ImmTACs barely triggered IFNγ secretion in response to endogenous levels of NY-ESO-1. Amounts of IFNγ were substantially smaller across the entire dose range, and the minimal ImmTAC concentration to trigger any IFNγ secretion was 1 nM (Fig. 1g, Supplementary Figure S1a). The E8 ImmTAC did not induce any IFNγ secretion anymore (Fig. 1g). Comparable results were observed for IFNγ secretion in response to the NY-ESO-1 knockdown Mel624 cells, with a 1 nM threshold for E42 (Supplementary Figure S1c). The amount of IFNγ secreted in response to the E42 ImmTAC, in contrast to all other ImmTACs, was independent of the antigen concentration (Fig. 1d), suggesting a loss of antigen specificity with the highest ImmTAC affinity for CD3ε.

Finally, we determined whether CD3ε engagement with ImmTACs synergizes with direct engagement of the α/β heterodimer of a TCR by peptide-MHC on the same T cell by comparing CD8^+^ CTL and 1G4 activation in response to 100 pM E28 ImmTAC. While killing by 1G4 CTL in response to E28 was slightly higher than that by CD8^+^ CTL, the difference did not reach statistical significance and is unlikely to be functionally relevant (Supplementary Figure S1d). IFNγ secretion was low with no substantial differences (Supplementary Figure S1e).

Together, these data establish that E28 and E42 ImmTACs at the most likely physiologically relevant concentration of 100 pM can trigger efficient cytolysis in response to limiting endogenous antigen presentation but without noticeable IFNγ secretion and irrespective of whether the CTL express a tumor-reactive TCR. Larger amounts of antigen enabled cytolytic responses by smaller amounts of ImmTAC, by ImmTACs with a weaker affinity for CD3ε and effective IFNγ secretion.

### The efficiency of cytotoxic T lymphocyte activation by immune-mobilizing monoclonal T cell receptors against cancer is comparable to that triggered by direct T cell receptor engagement by peptide-MHC

**Figure 2:**
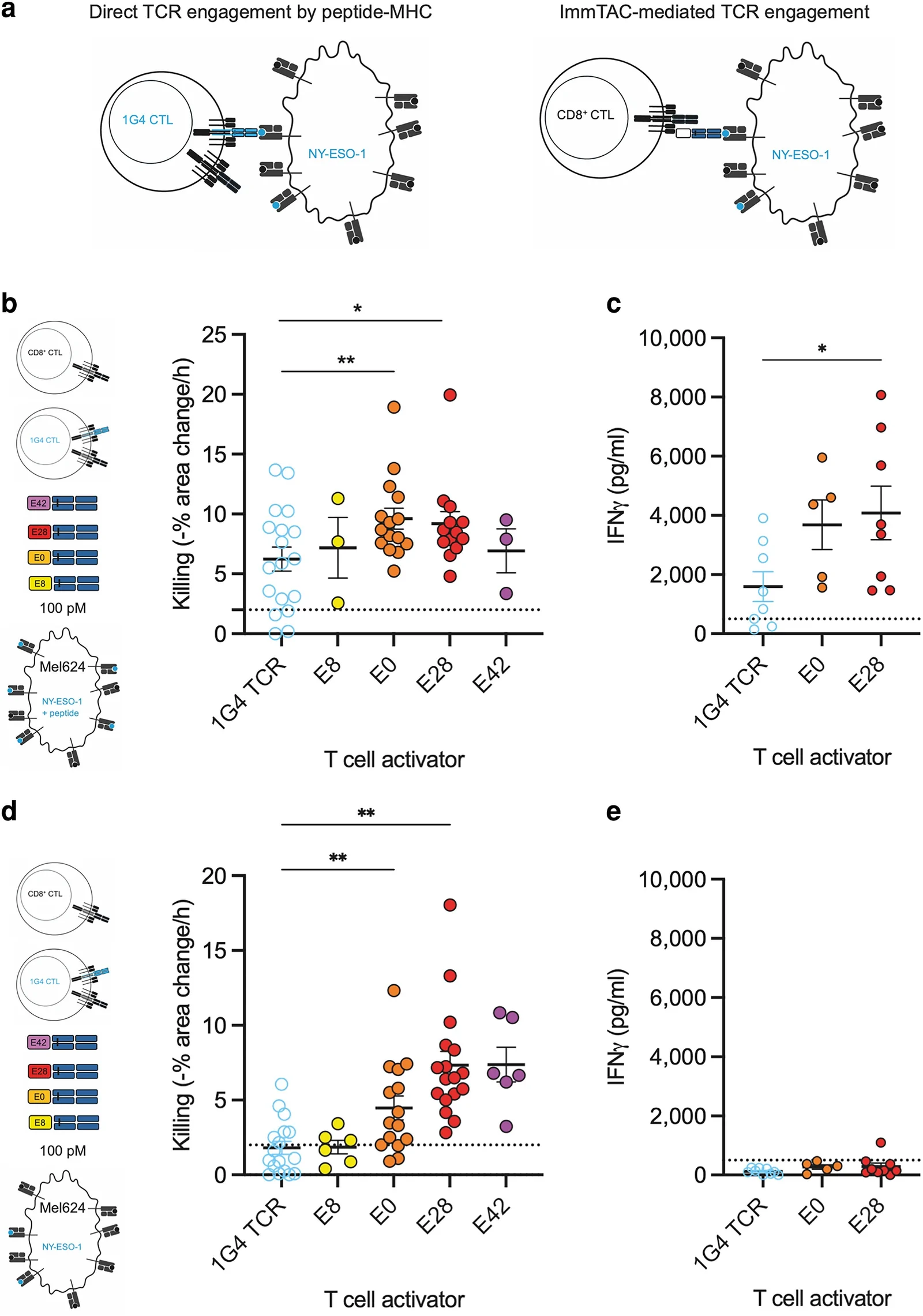
Efficiency of CTL activation by ImmTACs is comparable to that triggered by direct TCR engagement by peptide-MHC. **a** Graphical depiction of the direct activation of 1G4 CTL by NY-ESO-1_157–165_/HLA-A*02:01 versus the indirect activation of non-transduced CTL via ImmTACs recognizing the same NY-ESO-1_157–165_/HLA-A*02:01 complex. **b** Killing of Mel624 or A375 tumor target cells incubated with 2 µg/ml NY-ESO-1 peptide by 1G4 CTL or non-transduced CTL in the absence or presence of 100 pM of the given ImmTAC version; data pooled; mean ± SEM. 3–18 independent experiments. Statistical significance determined by paired one-way ANOVA. **c** IFNγ amounts in supernatants of Mel624 or A375 tumor target cells incubated with 2 µg/ml NY-ESO-1 peptide interacting with 1G4 CTL or non-transduced CTL in the absence or presence of 100 pM of the given ImmTAC version; data pooled; mean ± SEM. 5–8 independent experiments. Statistical significance determined by paired one-way ANOVA. **d** Killing of Mel624 or A375 tumor target cells by 1G4 CTL or non-transduced CTL in the absence or presence of 100 pM of the given ImmTAC version; data pooled; mean ± SEM. 6–17 independent experiments. Statistical significance determined by paired one-way ANOVA. **e** IFNγ amounts in supernatants of Mel624 or A375 tumor target cells interacting with 1G4 CTL or non-transduced CTL in the absence or presence of 100 pM of the given ImmTAC version; data pooled; mean ± SEM. 5–9 independent experiments. Statistical significance determined by paired one-way ANOVA. *P < 0.05, **P < 0.01.

To gauge the efficacy of ImmTACs in comparison to physiological CTL activation through direct TCR engagement by peptide-MHC, we used 1G4 CTL (Fig. 2a). In response to Mel624 cells incubated with saturating amounts of exogenous NY-ESO-1 peptide, CTL activation with 100 pM ImmTACs triggered cytolysis that was comparable if not superior to that of 1G4 CTL (Fig. 2b). IFNγ secretion triggered by 100 pM E28 ImmTAC was greater than that of 1G4 CTL (Fig. 2c). In response to endogenous NY-ESO-1 antigen processing, 100 pM E0, E28, and E42 again triggered comparable if not greater cytolysis (Fig. 2d). There was no IFNγ secretion under these conditions (Fig. 2e). In the interpretation of these data, it is important to consider that the 1G4 CTL express the 1G4 TCR in addition to endogenous TCRs and as stabilized with only a disulfide bridge in the constant domains [28], as used in adoptive cell therapy. This leads to comparatively low 1G4 TCR expression and less effective CTL activation through the 1G4 CTL than should be expected in CTLs only expressing a transgenic TCR [19]. The efficacy of CTL activation by direct engagement of a TCR by peptide-MHC is thus likely underestimated in Fig. 2. Moreover, other TCRs with higher or lower affinity for their cognate peptide-MHC will likely generate different dose–response curves. With these caveats in mind, the data still establish that CTL activation by the E28 and E42 ImmTACs at the concentration most likely to be physiologically relevant of 100 pM is comparable to that of CTL activation by direct engagement of the TCR by peptide-MHC.

### Immune-mobilizing monoclonal T cell receptors against cancer trigger tumor cell killing in a suppressive environment

**Figure 3:**
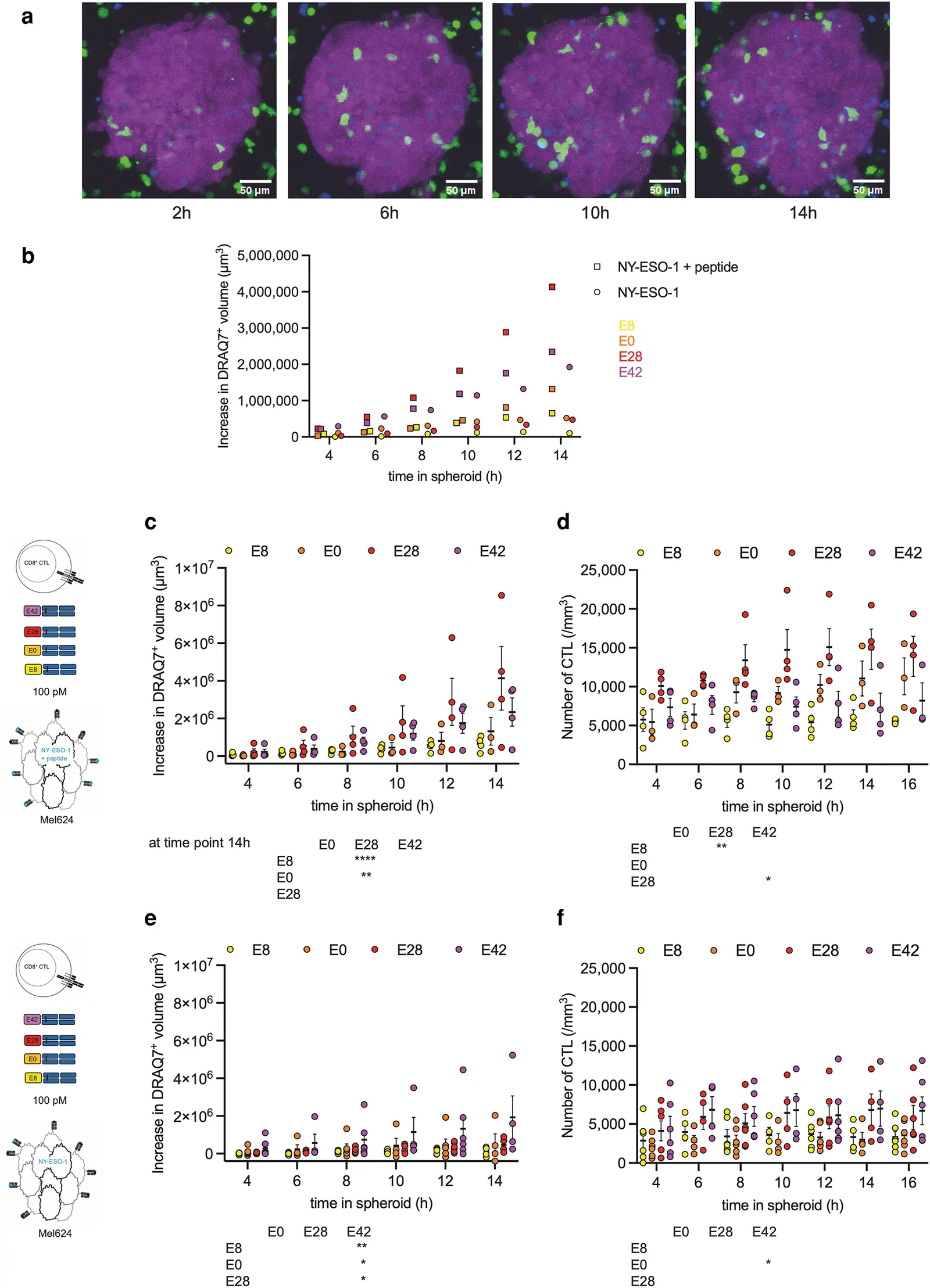
ImmTACs mediate target cell killing in tumor cell spheroids. a Representative spheroid killing data relative to the time of Matrigel embedding of Mel624 spheroids (purple) in the presence of exogenous NY-ESO-1 peptide with 100 pM E28, CD8^+^ CTL (green) and DRAQ7 staining (blue) to determine cell death. Maximum projection of 3D imaging data. Scale bar = 50 µm. **b, c** Non-transduced CTL cocultured with Mel624 spheroids incubated with 2 µg/ml NY-ESO-1 peptide. Each data point is an independent experiment (*n* = 3, 4) with a total of 8–11 spheroids analyzed per condition. (b) Spheroid death, as measured by the increase in DRAQ7^+^ spheroid volume, is shown. Statistical significance determined by paired mixed-effects analysis and given as differences between ImmTAC versions at 14 h and main effect of time in the tables at the right. Single-spheroid data are given in Supplementary Figure S2b. (c) SIL densities are shown with the mean ± SEM for the same experiments as in (b). Statistical significance determined by paired mixed-effects analysis and given as main effect of ImmTAC version in the table at the right. **d, e** Non-transduced CTL cocultured with Mel624 spheroids for 12 h with images acquired every 2 h or 4 h. Each data point is an independent experiment (*n* = 6) with a total of 14–16 spheroids analyzed per condition. (d) Spheroid death, as measured by the increase in DRAQ7^+^ spheroid volume, is shown. Statistical significance determined by paired mixed-effects analysis and given as differences between time points for E42 and main effect of ImmTAC version in the tables at the right. Single-spheroid data are given in Supplementary Figure S2d. (e) SIL densities are shown with the mean ± SEM for the same experiments as in (d). Statistical significance determined by paired mixed-effects analysis and given as main effect of ImmTAC version in the table at the right. *P < 0.05, **P < 0.01, ***P < 0.001, ****P < 0.0001.

In patients, ImmTACs must function in the suppressive TME. We use tumor cell spheroids to generate a suppressed CTL phenotype that is comparable to exhausted CTL in tumors [19] as further investigated for ImmTAC-treated CD8^+^ CTL below. Tumor cell killing is determined as the increase in dead spheroid volume in live spheroid imaging of Mel624 spheroids interacting with CTL [19] (Fig. 3a). In response to Mel624 spheroids incubated with saturating amounts of exogenous NY-ESO-1 peptide, 100 pM E28 and less so E42 triggered substantial time-dependent tumor cell killing (Fig 3b, c, Supplementary Figure S2a, b). This was accompanied by increased CTL infiltration into the spheroids upon treatment with E28 (Fig. 3d) and a time-dependent increase in the depth of infiltration (Supplementary Figure S2c). In response to endogenous NY-ESO-1 antigen processing by the Mel624 cells, only 100 pM E42 ImmTAC could trigger cytolysis (Fig. 3e, Supplementary Figure S2a, d). In comparison to spheroid incubation with exogenous peptide, spheroid infiltration was reduced in response to E0 and E28, consistent with the lack of killing (Fig. 3f). Infiltration depth was largely unchanged (Supplementary Figure S2e). The differential behavior of the E28 and E42 ImmTACs, E28 being more effective at the saturating peptide concentration, E42 at the endogenous one, is similar to their effects on IFNγ secretion in the 2D killing assays (Fig. 1d, e, g). These data are consistent with data on agonist antibodies showing optimal triggering of effector function at an intermediate level of receptor engagement [27]. The extent of killing triggered by the ImmTAC E0 was comparable to killing triggered by direct TCR engagement with peptide-MHC in 1G4 CTL (Supplementary Figure S3). Together, these data establish that ImmTACs at the concentration most likely to be physiologically relevant of 100 pM can trigger CTL infiltration into tumor cell spheroids leading to target cytolysis dependent on anti-CD3 affinity, particularly in response to limiting endogenous amounts of antigen. CTL infiltration into spheroids and tumor cell killing were only partially related. Some infiltration occurred without triggering cytolysis. Going forward, based on the data from Figs 1–3, we will focus on ImmTAC E28 as the most effective ImmTAC triggering CTL activation while maintaining consistent antigen-dependence and occasionally include E0 to determine the role of different on/off rates.

### Immune-mobilizing monoclonal T cell receptors against cancer trigger cytotoxicity in suppressed cytotoxic T lymphocytes and don’t induce suppression thereof

**Figure 4:**
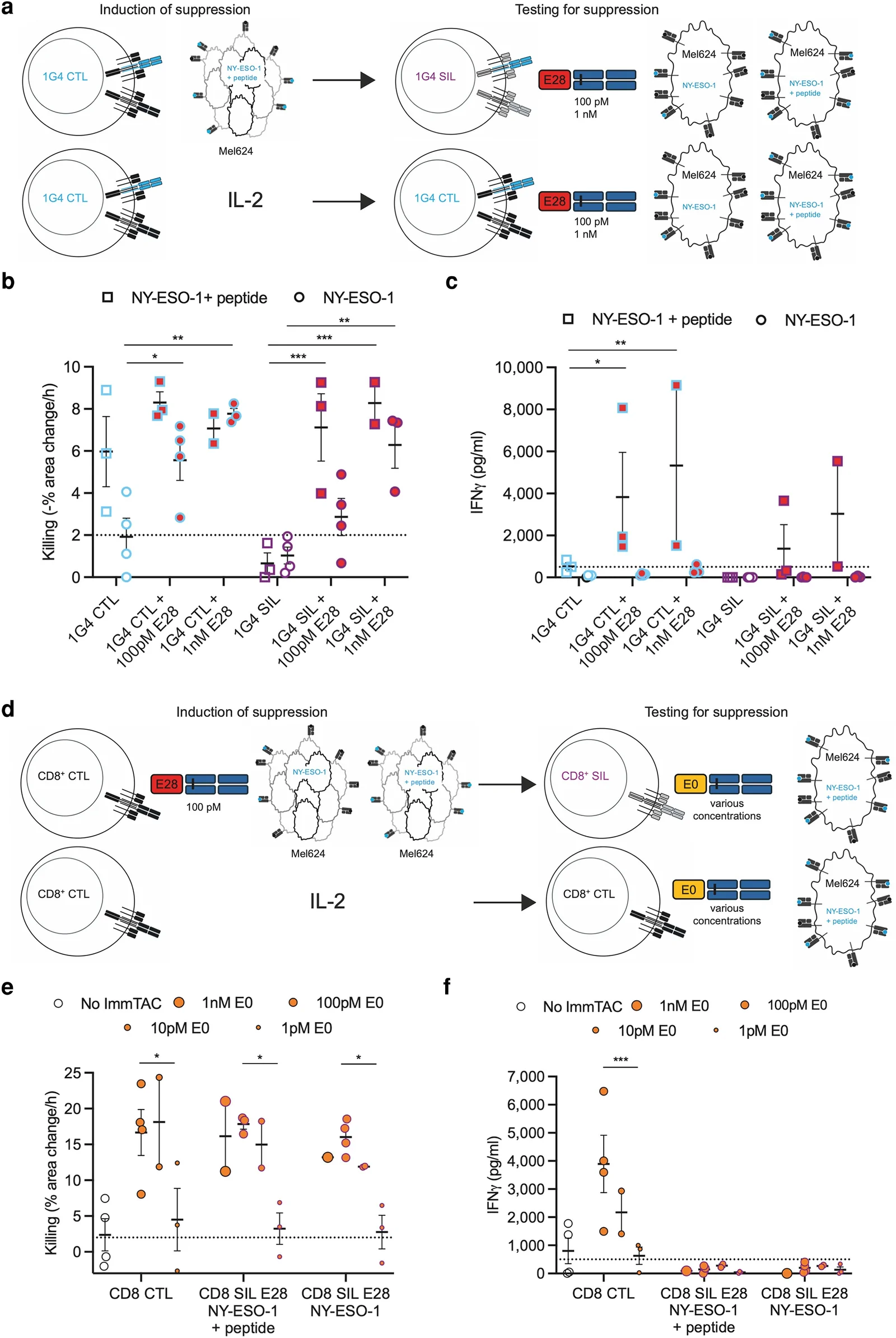
ImmTACs can redirect suppressed CTL to kill targets but do not rescue IFNγ secretion. **a** Graphical depiction of the induction of 1G4 CTL suppression and the subsequent experiment to assess activation of suppressed 1G4 SIL by ImmTAC (top row) and of the corresponding control 1G4 CTL experiment (bottom row). **b**, **c** Killing (b) of Mel624 tumor target cells incubated with 2 µg/ml NY-ESO-1 peptide or not by 1G4 CTL or 1G4 SIL in the absence or presence of the indicated concentration of the E28 ImmTAC and (c) IFNγ amounts in the corresponding supernatants; mean ± SEM. 4–6 independent experiments. Statistical significance determined by paired Mixed-effects analysis. **d** Graphical depiction of the experiment to test induction of suppression by ImmTAC-mediated CTL activation in spheroids and the subsequent experiment to test for such suppression (top row) and of the corresponding non-suppressed control experiment (bottom row). **e, f** Killing (e) of Mel624 tumor target cells incubated with 2 µg/ml NY-ESO-1 peptide by non-transduced CTL, previously incubated or not in spheroids incubated with 2 µg/ml NY-ESO-1 peptide or not, in the presence of 100 pM E28 ImmTAC in the absence or presence of the indicated concentration of the E0 ImmTAC and (f) IFNγ amounts in the corresponding supernatants; mean ± SEM. 1–4 independent experiments. Statistical significance determined by paired Mixed-effects analysis. *P < 0.05, **P < 0.01, ***P < 0.001.

Suppressed CTL can be re-isolated from tumor cell spheroids as spheroid-infiltrating lymphocytes (SIL) to allow further investigation of their properties. To corroborate and extend the in-spheroid ImmTAC killing data (Fig. 3), we incubated 1G4 CTL with Mel624 tumor cell spheroids to induce suppressed 1G4 SIL, re-isolated them from the spheroids and determined the ability of ImmTACs to activate these suppressed cells (Fig. 4a). In the absence of ImmTAC in response to direct 1G4 TCR engagement by NY-ESO-1_157–165_/HLA-A*02:01, 1G4 CTL as the control cells that had not been incubated with spheroids, killed Mel624 target cells. However, 1G4 SIL could not, whether exogenous NY-ESO-1 peptide was added or not (Fig. 4b), corroborating the suppressed state of the 1G4 SIL. Conversely, 100 pM E28 triggered cytolysis of Mel624 cells by 1G4 SIL in the presence of exogenous NY-ESO-1 peptide that was comparable to control 1G4 CTL that had not been incubated with spheroids (Fig. 4b). Even when relying on the limiting endogenous NY-ESO-1 antigen processing, 100 pM E28 induced some Mel624 target cell killing by 1G4 SIL; 1 nM E28 elicited cytolysis comparable to that of control 1G4 CTL (Fig. 4b). Consistent with the in-spheroid killing data, E28 ImmTAC thus could trigger tumor target cell killing in suppressed CTL, at a low level in response to endogenous antigen, efficiently at higher amounts of antigen or ImmTAC.

However, E28 was less effective at inducing IFNγ secretion by suppressed CTL. In response to endogenous antigen processing, even at a 1 nM concentration, E28 did not induce substantial IFNγ secretion by 1G4 CTL or 1G4 SIL (Fig. 4c). In one of two experimental repeats and at saturating amounts of exogenous NY-ESO-1 peptide, E28 triggered some IFNγ secretion, albeit less than in control 1G4 CTL (Fig. 4c). Thus, at the physiologically most relevant low antigen and ImmTAC concentrations, the E28 ImmTAC could not induce IFNγ secretion in suppressed CTL.

Antigen engagement by 1G4 CTL in spheroids overnight triggers suppression [19]. We, therefore, wanted to investigate whether comparable ImmTAC engagement in spheroids also does so. We incubated CD8^+^ CTL in Mel624 spheroids with 100 pM E28 in the presence or absence of exogenous NY-ESO-1 peptide and re-isolated the T cells as “CD8 SIL E28 NY-ESO-1 + peptide” and “CD8 SIL E28 NY-ESO-1,” respectively. This spheroid incubation is where any ImmTAC-mediated suppression would be induced. To test whether the suppression has indeed been induced or not, we then compared the activation of these SIL to CD8^+^ CTL that had not been incubated with spheroids, fully active CTL. In these activation assays, we varied antigen amounts by the presence or absence of exogenous NY-ESO-1 peptide, and we stimulated the T cells with increasing concentrations of E0 (from 1 pM to 1 nM) (Fig. 4d). We used E0 instead of E28 in this suppression test as the weaker stimulus provided by E0 (Fig. 1f) should make it easier to detect any form of suppression. E0 ImmTAC elicited CD8 SIL killing of targets comparable to control CD8 CTL (Fig. 4e), establishing the absence of induction of suppression of cytolysis. However, the overnight incubation of CD8^+^ CTL with ImmTAC in spheroids prevented the CTL from subsequently secreting IFNγ, even in the presence of exogenous peptide (Fig. 4f). Thus, inducing suppression and overcoming it by ImmTACs differed by CTL effector function: ImmTAC could not only rescue cytolysis in suppressed CTL (Fig. 4b) but did so without inducing a suppressed state during an overnight incubation (Fig. 4e). In contrast, such incubation induced suppression of IFNγ secretion, and ImmTACs could not trigger IFNγ secretion in suppressed CTL.

To verify that TCR engagement by antigen in the spheroids is required for the induction of suppression, we incubated CD8^+^ CTL with Mel624 spheroids. Because of the diverse TCR repertoire of the CD8^+^ CTL, antigen-specific TCR engagement will be minimal. To prevent CTL reversion to a resting phenotype, spheroids were incubated with IL-2 (Supplementary Figure S4a). We then tested for the induction of suppression similar to Fig. 4d. CD8^+^ SIL after they had been incubated in the absence of antigen with spheroids killed Mel624 target cells in response to 100 pM E0 and secreted IFNγ as well as control CD8^+^ CTL (Supplementary Figure S4b, c), establishing absence of suppression. The induction of CTL suppression in spheroids thus required TCR engagement.

To more broadly address the ability of ImmTACs to overcome suppression, we investigated an alternate means of inducing CTL suppression: coculture of tumor target cells with fibroblasts (Supplementary Figure S4d) to mimic cancer-associated fibroblasts [29]. When Mel624 cells were cocultured with MRC-5 fibroblasts for 16 h before the killing assay, 100 pM E28 ImmTAC no longer elicited CD8^+^ CTL killing of Mel624 cells presenting endogenous levels of NY-ESO-1 peptide (Supplementary Figure S4e). MRC-5 fibroblasts were not killed in the same experiments despite low-level endogenous expression of NY-ESO-1 (not shown and Supplementary Figure S4f), removing fibroblast death as a potential confounder of the experiment. IFNγ secretion under these conditions was too low to allow a meaningful analysis. These results suggest that in a complex TME with a dense fibroblast network, ImmTAC may not be sufficient to rescue CTL suppression and redirect T cells to kill target tumor cells.

### Immune-mobilizing monoclonal T cell receptors against cancer effectively enhance the subcellular polarization of cytotoxic T lymphocytes interacting with tumor target cells

**Figure 5:**
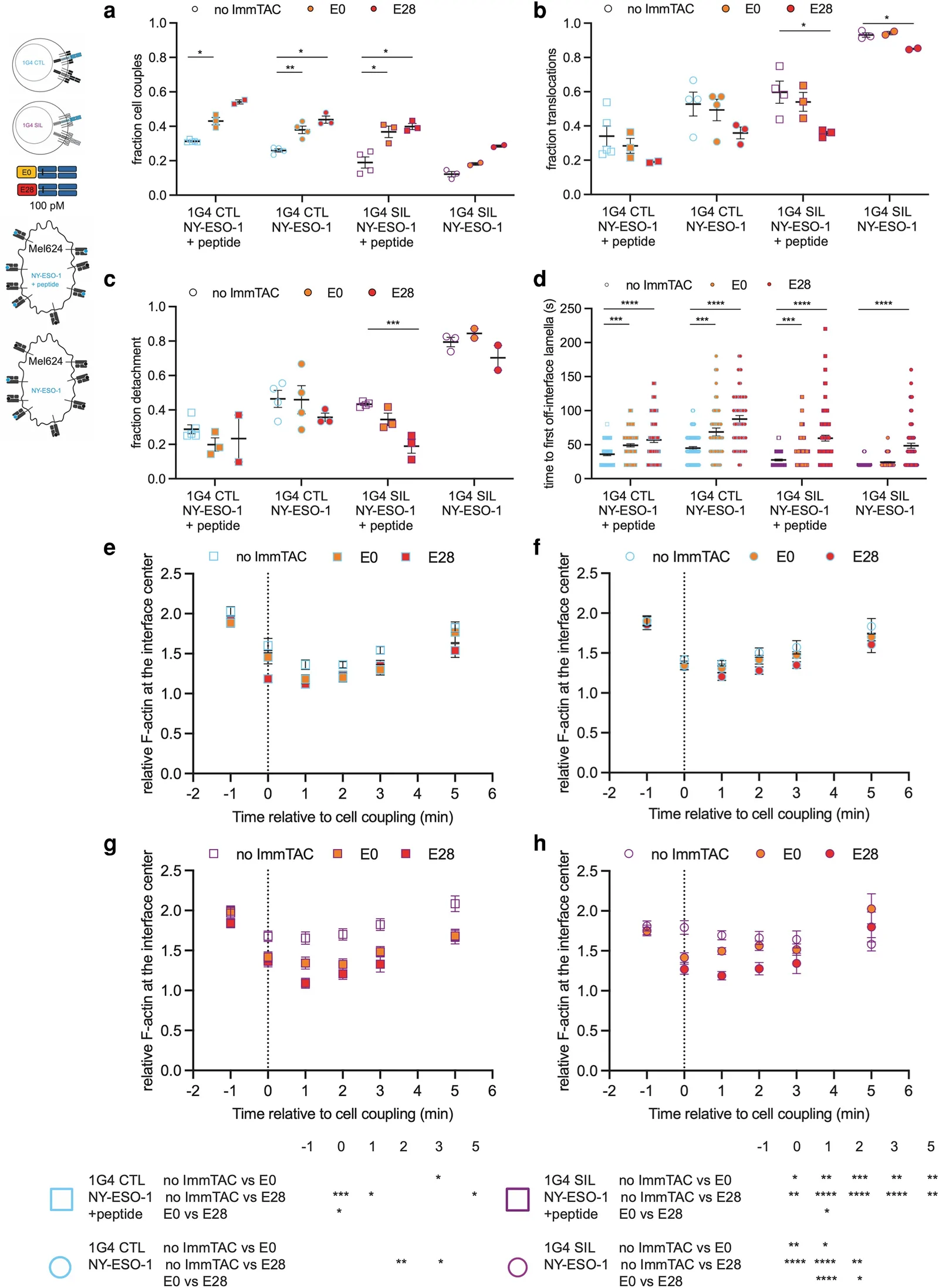
ImmTACs effectively induce cytoskeletal polarization of CTLs interacting with tumor target cells. **a–d** Characterization of cell morphology in the interaction of 1G4 CTL or SIL with Mel624 cells in the presence or absence of 2 µg/ml NY-ESO-1 agonist peptide and 100 pM of the indicated ImmTAC; mean ± SEM. (a) Fraction of 1G4 CTL or SIL converting a target cell contact into a tight cell couple. Each symbol is an imaging run. (b) Fraction of CTL with a translocation. Each symbol is an imaging run. (c) Fraction of CTL with detachment. Each symbol is an imaging run. (d) Time from tight cell couple formation to first off-interface lamella. Each symbol is a cell couple. 2–5 independent experiments. Statistical significance determined by one-way ANOVA (a–c) and Kruskal–Wallis test (d). **e–h** F-actin accumulation at the center of the interface between 1G4 CTL (e, f) and 1G4 SIL (g, h) expressing F-tractin-GFP and Mel624 cells in the presence (e, g) or absence (f, h) of NY-ESO-1 agonist peptide and 100 pM of the indicated ImmTAC relative to F-actin in the entire cell and to the time of tight cell coupling; mean ± SEM. Pooled data from 2 to 3 independent experiments. Single-cell data in Supplementary Figure S5e-h. Statistical significance determined by two-way ANOVA and given in the table at the bottom right. *P < 0.05, **P < 0.01, ***P < 0.001, ****P < 0.0001.

The execution of target cell killing by CTL requires the formation and effective maintenance of CTL target cell couples [15, 17, 19]. We therefore investigated whether ImmTACs can support such subcellular polarization of both CTL and SIL at different levels of antigen. We determined six elements of CTL tumor cell couples: (i) the formation of a tight cell couple upon initial CTL tumor cell contact as a measure of CTL-perceived initial stimulus strength (Supplementary Figure S5a), (ii) the time of formation of the first off-interface lamella, (iii) frequency and time of translocation of the CTL over the tumor cell surface (Supplementary Figure S5b), (iv) “detachment,” the complete repolarization away from the tumor cell (Supplementary Figure S5c), as measures of inefficient maintenance of CTL-tumor cell couples, (v) F-actin clearance at the interface center (Supplementary Figure S5a) is associated with efficient delivery of lytic granules [15] and, (vi) F-actin accumulation at the entire interface (Supplementary Figure S5a-c) is required for interface formation and maintenance. Incubation with ImmTACs consistently led to more stable cell couples in the form of enhanced cell couple formation (Fig. 5a), reduced translocations and detachments (Fig. 5b, c, only for E28 and significantly only for 1G4 SIL), delayed formation of the first off-interface lamellae, and delayed translocations (Fig. 5d, Supplementary Figure S5d). As previously shown, clearance of F-actin from the interface center was less effective in the suppressed SIL than in active CTL (Fig. 5e–h, Supplementary Figure S5e-h) [19]. ImmTACs E0 and E28 enhanced F-actin clearance under all conditions. The enhancement upon ImmTAC treatment was more pronounced in SIL than in CTL, leading to comparable levels of clearance under all conditions (Fig. 5e–h, Supplementary Figure S5e–h). Overall interface F-actin accumulation didn't differ (Supplementary Figure S6). E28 was consistently more effective than E0. These polarization data closely match the ability of ImmTACs to induce effector function (Figs 1–3). High frequencies of translocation and detachment are associated with an inability of T cells to kill [19]. While in response to saturating amounts of exogenous antigen 100 pM E28 could reverse elevated frequencies of translocation and detachment in SIL to levels observed in CTL activation, in response to endogenous antigen 100 pM E28 reduced translocation frequencies only slightly, without reaching levels seen in CTL. These data are consistent with the limited ability of E28 to induce cytolysis in SIL in response to endogenous antigen (Figs 3e, 4b). Together, these data establish that ImmTACs, similar to direct TCR engagement by peptide-MHC, support a key cellular mechanism of target cell lysis, the effective subcellular polarization of CTL as required for the release of lytic granules.

### Immune-mobilizing monoclonal T cell receptors against cancer trigger effective calcium signaling but no short-term changes in coregulatory receptor expression

**Figure 6:**
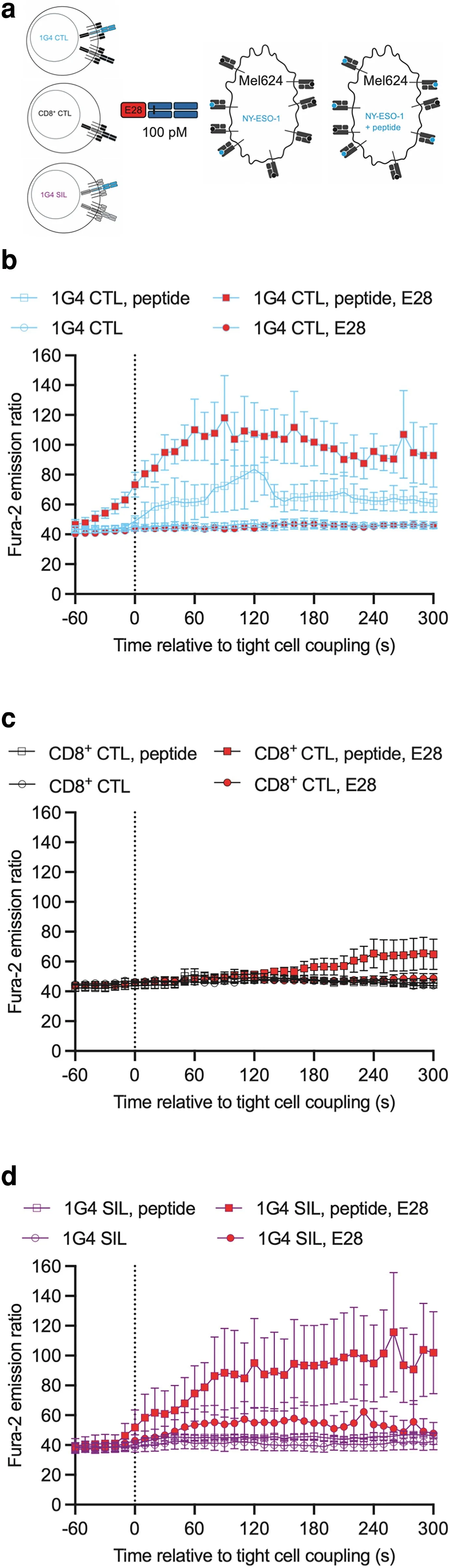
E28 ImmTAC triggers the elevation of the CTL cytoplasmic calcium concentration more effectively than direct engagement of a TCR by peptide-MHC. **a** CTL and target cells used in (b–d). **b–d** Ratio of the Fura-2 emission upon excitation at 340 nm over 380 nm of 1G4 CTL (b), CD8^+^ CTL (c), or 1G4 SIL (d) activated by Mel624 cells in the presence or absence of 2 µg/ml NY-ESO-1 agonist peptide and 100 pM E28; mean of independent experiments ± SEM. 3–4 independent experiments. Statistical significance determined by paired mixed-effects analysis and given in Supplementary Figure S7a. Single-cell data are given in Supplementary Figure S7b-d.

The elevation of the cytoplasmic calcium concentration is a key proximal signaling event in CTL activation that closely correlates with IFNγ secretion [18]. Using the calcium-sensitive dye Fura-2, we determined calcium signaling in 1G4 CTL, CD8^+^ CTL, and 1G4 SIL in response to 100 pM E28 as compared to direct engagement of the 1G4 TCR by peptide-MHC (Fig. 6a). Measurable calcium signaling in 1G4 CTL required the incubation of Mel624 cells with exogenous NY-ESO-1 peptide. 100 pM E28 further enhanced calcium signaling triggered by direct engagement of the 1G4 TCR with peptide-MHC (Fig. 6b, Supplementary Figure S7a). As expected, no calcium signaling was observed in CD8^+^ CTL in the absence of the E28 ImmTAC (Fig. 6c, Supplementary Figure S7b). Additionally, calcium signaling triggered by Mel624 cells in the presence of exogenous NY-ESO-1 peptide and 100 pM E28 was smaller in CD8^+^ CTL than in 1G4 CTL (Fig. 6b, c). These data suggest that the E28 ImmTAC is more effective in triggering calcium signaling when acting in parallel to direct TCR engagement by peptide-MHC rather than in isolation. Such enhanced signaling did not translate into enhanced effector function (Supplementary Figure S1d, e) as further discussed below. In 1G4 SIL, no calcium signaling was detected in the absence of E28, whether the Mel624 cells were incubated with exogenous NY-ESO-1 peptide or not, consistent with the suppressed state of the 1G4 SIL (Fig. 6d, Supplementary Figure S7c). E28 could trigger effective calcium signaling in the presence of exogenous NY-ESO-1 peptide, consistent with its ability to overcome 1G4 SIL suppression under these conditions (Fig. 4b).

To determine whether ImmTAC treatment on the overnight time scale of our experiments changes protein expression, we determined the expression of 17 T cell markers focused on coregulatory receptors in the interaction of CD8^+^ CTL with Mel624 cells incubated with exogenous NY-ESO-1 peptide in the presence of 100 pM E28 for 4 and 16 h. No substantial changes occurred, neither in the percentage of CD8^+^ CTL positive for a given marker, nor in the mean fluorescence intensity (Supplementary Figure S8, S9a, b). The lack of substantial changes upon ImmTAC treatment is confirmed in a cluster analysis (Supplementary Figure S9c–e). A small fraction of the CTL displayed a more exhausted phenotype characterized by higher expression of inhibitory receptors and lower expression of TCF1 and SLAMF6. However, this population was restricted to strongly stimulated CTL, CTL in the presence of IL-2 or ImmTAC for 4 h. On the time scale of the execution of CTL effector function investigated here, effects of ImmTACs thus were predominantly posttranslational.

## Discussion

One ImmTAC, Tebentafusp, that recognizes a peptide derived from gp100 as presented by HLA-A*02:01, is clinically approved for the treatment of metastatic uveal melanoma [10, 11]. However, mechanisms by which ImmTACs activate CTLs remain partially unresolved. Here we show that a model ImmTAC recognizing an NY-ESO-1-derived peptide presented by HLA-A*02:01 is a powerful inducer of the cytotoxicity of fully active CTL *in vitro* across varying antigen amounts in the tumor target cells, ImmTAC concentrations and ImmTAC affinities for CD3ε. Induction of IFNγ secretion remained limited across all those conditions. The ability to induce cytolysis extended to CTL suppressed by interaction with tumor cell spheroids, albeit becoming limited to ImmTACs with the highest affinity for CD3ε. ImmTACs thus could overcome suppression of CTL cytotoxicity. ImmTACs consistently enhanced the ability of CTL to form and maintain stable cell couples with tumor target cells as a likely mechanism of their efficient induction of CTL cytotoxicity without substantial changes in the expression of CTL activation and suppression markers.

ImmTACs use an anti-CD3ε scFv antibody fragment to trigger TCR signaling. In the use of agonist antibodies, varying the antibody affinity commonly results in a bell-shaped response curve where both low and high affinities trigger suboptimal cellular effector function [27, 30]. In this paper, we performed experiments using ImmTAC molecules engineered with a series of anti-CD3ε scFv antibody fragments of varying affinity. We observed that in IFNγ secretion, in response to NY-ESO-1 peptide-pulsed targets, a similar bell-shaped affinity response curve exists for ImmTACs, with the most potent induction of IFNγ secretion at an anti-CD3ε affinity of 35 nM [13], the affinity of the E0 and E28 ImmTACs. However, when we reduced the stimulus strength to endogenous NY-ESO-1 antigen processing and used fully active or suppressed CTL as effectors, the affinity response curve shifted. Under these antigen-limiting conditions, T cell activation was most effective using the highest affinity anti-CD3ε scFv antibody fragment, E42. However, at this highest anti-CD3ε affinity, IFNγ secretion became independent of antigen amounts. A similar shift in the affinity response curve was found *in vitro* when reducing the affinity of the TCR moiety of the ImmTAC for MHC I/peptide [13]. Adjusting the anti-CD3ε affinity of an ImmTAC in response to antigen amounts determined in a tumor biopsy thus may be an effective strategy to adapt the ImmTAC to the physiological circumstances of therapeutic application. Potential losses in specificity with increasing affinity [13] need to be considered in doing so. These considerations should also apply to the design of new TCR engagers [21] based on synthetic peptide-MHC binders [21–23] which currently have a comparatively low affinity for the peptide-MHC complex (in the low nM range) that may require compensation by a higher affinity anti-CD3ε scFv antibody fragment.

In therapeutic settings, ImmTACs must function under conditions of CTL suppression in the TME. We have used tumor cell spheroids here to reflect such suppression. In the presence of a high amount of antigen, the E28 ImmTAC with an intermediate affinity for CD3ε effectively elicited killing of tumor target cells in spheroids and upon isolation of suppressed T cells from spheroids. Using the high-affinity E42 ImmTAC, cytolytic activity was observed against Mel624 cells presenting low levels of endogenous NY-ESO-1 peptide, again linking antigen dose to ImmTAC CD3ε affinity. Antigen engagement under suppressive conditions triggers CTL exhaustion. ImmTACs were not only able to overcome CTL suppression in a spheroid system, but also did so without inducing suppression of the cytolytic ability of CTL. However, ImmTACs could not overcome suppression of IFNγ secretion, and after ImmTAC incubation in spheroids, SIL IFNγ secretion was completely suppressed. These data establish a cytolysis versus IFNγ split suppression by ImmTACs, strongly focusing expected *in vivo* effector function of ImmTACs in the suppressive TME on cytolysis. Phenotyping experiments will need to determine the molecular characteristics of this ImmTAC-induced split suppression. A requirement for stronger stimuli to trigger IFNγ secretion versus cytolysis that may underlie this split has been described and discussed before by us and others [19, 31]. As an additional element of CTL suppression, killing of target cells was suppressed by stromal cells even in active CTL, possibly limiting ImmTAC efficacy in stroma-rich tumors.

In the extrapolation of *in vitro* data to ImmTAC function in a therapeutic setting, we have not considered CD4^+^ T cells. *In vitro* ImmTACs can trigger target cell killing and cytokine secretion by effector CD4^+^ T cells [32]. This may provide an alternative source of IFNγ in patients. The effect on regulatory T cells has not been determined either, but previous work showed that *in vitro* ImmTAC-triggered CD8^+^ T cell function is insensitive to the presence of regulatory T cells [32]. For a more comprehensive extrapolation of mechanisms of ImmTAC function *in vivo*, future work will need to focus on CD4^+^ T cells, helper and regulatory, under suppressive conditions and on CD8^+^ and CD4^+^ T cells in conjunction.

Activating a T cell by replacing the engagement of its TCR by peptide-MHC complexes with synthetic approaches can lead to substantial alterations in T cell signaling. For example, proximal T cell signaling triggered by chimeric antigen receptors is substantially less efficient [20]. The total size of a receptor-ligand complex at the interface between a T cell and an activating antigen-presenting cell is important for signaling efficiency [33]. This could be relevant here, as the total size of a peptide-MHC-ImmTAC-CD3ε complex might be larger than that of a peptide-MHC-TCRα/β complex. When assayed as the ability to trigger cytolysis and IFNγ secretion, T cell activation by ImmTACs at physiologically realistic concentrations displayed a comparable peptide sensitivity and extent of effector function to T cell activation by direct TCR engagement with peptide-MHC. Moreover, ImmTACs triggered the same effective execution of a series of CTL subcellular polarization steps required for efficient cytolysis as direct TCR engagement. ImmTACs thus closely mimic physiological T cell activation. There was one notable difference. Under the limiting T cell activation conditions investigated here, the elevation of the intracellular calcium concentration triggered by direct TCR engagement was inefficient. Calcium signaling triggered by ImmTAC in CTL not expressing a tumor antigen-reactive TCR was equally inefficient. However, calcium signaling triggered by ImmTAC in 1G4 CTL was substantially elevated, in particular in SIL. Such elevation, however, did not correlate with more efficient CTL effector function. Calcium signaling is only one of many signaling pathways triggered during T cell activation. We have previously shown that cytolysis is more closely associated with F-actin dynamics than with calcium signaling [18], consistent with our killing data. Signaling processes that link the induction of calcium signaling by E28 ImmTAC as opposed to that by direct TCR engagement by peptide-MHC to IFNγ secretion could operate at the level of transcription or vesicular secretion, as to be determined. In combination, our data suggest that TCR engagement at a single point of the TCR complex, be it the α/β dimer or CD3ε, is comparably efficient. However, engagement of the complex at two points, α/β dimer plus CD3ε, be it on the same TCR molecule or different TCR molecules at the same cellular interface, can trigger more efficient proximal signaling. We speculate that similar engagement of coregulatory receptors in these two scenarios prevents the more efficient dual TCR engagement from triggering stronger effector function. The molecular mechanism of these signaling differences needs to be resolved in the future. However, the strong similarity in the sensitivity and cellular mechanism of T cell activation by ImmTACs in comparison to direct TCR engagement suggests that mechanisms of the physiological regulation of T cell activation, e.g. control by coregulatory receptors, may apply to T cell function in patients treated with ImmTACs. ImmTACs thus may benefit from combination with therapies that have already proven effective in the activation of endogenous T cells, such as checkpoint blockade.

## Materials and methods

### Human blood samples

Blood buffy coats from anonymous donors were purchased from NHS-BT with human work approved by the London-Riverside Research Ethics Committee under reference number 20/PR/0763.

### Antibodies and proteins

Antibodies are listed in the Supplementary Materials. ImmTAC proteins with varying anti-CD3ε scFv effector domains were expressed in the BL21 (DE3) Rosetta pLysS strain, refolded from inclusion bodies and purified as previously described [13, 34]. Purity was checked by reducing and nonreducing SDS-PAGE, and concentrations were assessed by A280 measurement and extinction coefficients derived from sequence by the inbuilt DNAdynamo algorithm.

### Human cell culture

Human cell culture was executed as previously described [19] with minor adjustments. Human A375 melanoma (RRID:CVCL_0132) and Mel624 melanoma (RRID:CVCL_8054) cells transfected to express mCherry or tdTomato [19, 35] were maintained in high-glucose DMEM or RPMI-1640, respectively, with 10% FBS, 2 mM glutamine, 1 mM pyruvate (DMEM/RPMI complete medium) supplemented with 250 μg/ml hygromycin. MRC-5 human embryonic lung fibroblasts (RRID:CVCL_0440) were transfected to express GFP and maintained in DMEM complete medium.

The 1G4 TCR, stabilized with an additional disulfide bridge in the constant domains [28], was expressed in primary human T cells using a pHR_SFFV-based lentiviral vector (RRID_Addgene79121) with an expression cassette of alpha chain-P2A-beta chain-P2A-GFP (as a sorting marker) or F-tractin-GFP (for F-actin imaging) [19]. Primary human CTL were derived from blood buffy coats, lentivirally transduced for the expression of the 1G4 TCR, and maintained in cell culture as described in the Supplementary Materials.

### Spheroids and spheroid-infiltrating lymphocytes

Experiments with spheroids and SIL were executed as previously described [19] with minor adjustments. Mel624 tdTomato cells were resuspended at a concentration of 1 × 10^5^ cells/ml, mixed with Matrigel (Corning) at 4°C, seeded in a 24-well plate at a final concentration of 500 cells per Matrigel dome, and left to solidify for 10 min at 37°C. 2 ml RPMI complete medium was added to each well and cells incubated at 37°C for 11 days.

For the generation of SIL, each Matrigel dome was washed twice in PBS and incubated for 1 h with 1 ml of Cell Recovery Solution (Corning) at 4°C. Spheroids were collected in a 15 ml Falcon tube and pulsed with NY-ESO-1 peptide at a final concentration of 2 μg/ml or with 100 pM of the E28 ImmTAC for 1 h each or left unpulsed. Spheroids were re-embedded in Matrigel together with 5 × 10^6^ 1G4 or non-transduced CD8^+^ CTL per Matrigel dome. Matrigel domes were dissolved for analysis of spheroid-infiltrating T cells after 16 h: Spheroids were washed twice in PBS and incubated with 1 ml of Cell Recovery Solution (Corning). Spheroids were collected, washed through a 40 μm sieve and then disaggregated to retrieve T cells in 1 ml of 10% FBS in PBS with 1 mM CaCl_2_ and 0.5 mM MgCl_2_ for immediate FACS sorting.

To determine CTL killing and infiltration into spheroids by live cell imaging, spheroids were dissociated from Matrigel and resuspended in fresh Matrigel at a concentration of ∼8 spheroids/µl. 50 µl of the spheroid-Matrigel suspension was separated into Eppendorf tubes, followed by the addition of 500 000 CTL per tube. 50 µl of Matrigel, containing spheroids and T cells, was plated into each well of a 24-well black, glass-base, sterile Sensoplate^TM^. After Matrigel had set, 1 ml of Fluorobrite medium (ThermoFisher) was added to each well, containing 1.5 µM DRAQ7 viability dye. Images were acquired every 2 h post-plating CTL with spheroids in 3 µm z steps from the bottom of the spheroid to its widest point, usually 40 steps, for 16 h using a Leica SP8 AOBS confocal microscope with a 10× HC PL Fluotar lens (NA = 0.3). Spheroids, SIL, and dead cells were segmented based on red, green, and purple fluorescence, respectively, using a custom Fiji ImageJ script as described before [36]. The increase in DRAQ7 volume measures overwhelmingly tumor cell death, as confirmed by lack of overlap with the green CTL fluorescence. Distance of each T cell from the spheroid surface was automatically calculated using the script.

### Cytolysis and IFNγ secretion

Cytolysis and IFNγ secretion were determined as previously described [19] with minor adjustments. For imaging-based cytotoxicity assays, the IncuCyte™ Live Cell analysis system and IncuCyte™ ZOOM software (Essen Bioscience) were used to quantify target cell death. 1 × 10^6^ Mel624 or A375 cells transfected to express the fluorescent protein mCherry or tdTomato were either untreated or pulsed for 1 h with 2 µg/ml of HLA-A*02:01 NY-ESO-1_157–165_ (SLLMWITQC), HLA-A*02:01 MART-1_26–35_ with the A27L mutation (“ELA”) (ELAGIGILTV) or the indicated concentration of an ImmTAC. Cells were suspended in 5 ml Fluorobrite medium (ThermoFisher) with 10% FBS, 2 mM L-glutamine, and 50 µM 2-mercaptoethanol to a concentration of 10 000 cells/50 μl (60–80% confluence). MRC5 cells were transfected to express GFP. Cells were plated in each well of a 384-well Perkin-Elmer plastic-bottomed plate and incubated for 4 h to adhere. For Mel624 MRC5 cocultures, cells were seeded at a 1:1 ratio and incubated for 16 h. A total of 40 000 CTL, non-transduced or that had been FACS sorted for GFP, were added per well to the plate in 50 μl Fluorobrite medium, yielding a 4:1 effector-to-target ratio. Images were taken every 15 min for 14 h at 1600 ms exposure using a 10× (NA = 0.3) lens. The total red object (adherent mCherry/tdTomato target cell) area (µm^2^/well) was quantified at each time point. To determine MRC5 killing, the green object area was quantified. A change in red/green area was determined as the linear gradient of the red/green object data at its steepest part for 6 h. The CTL killing rate was calculated as the difference in such change in red/green area in the presence and absence (to account for tumor cell proliferation) of CTL in the same 6 h time window and displayed as the percentage area change per 1 h. The determination of IFNγ amounts in the killing assay supernatant is described in the Supplementary Materials.

### Imaging of cytotoxic T lymphocytes and spheroid-infiltrating lymphocytes

Imaging of CTL and SIL was executed as previously described [19]. Prior to imaging, CTL and SIL were resuspended in “imaging buffer” (10% FBS in PBS with 1 mM CaCl_2_ and 0.5 mM MgCl_2_). As target cells, 1 × 10^6^ Mel624 melanoma cells were pulsed with the indicated peptide (as listed above) at a final concentration of 2 µg/ml for 1 h or left unpulsed. Glass-bottomed, 384-well optical imaging plates (Brooks Life Science Systems) were used for all imaging experiments. Imaging of F-actin distributions and CTL morphology was done at 37°C using an Olympus IXplore SpinSR confocal system incorporating a Yokogawa CSU-W1 SoRa spinning disk. A 60× oil-immersion lens (NA = 1.5) was used for all imaging experiments, unless otherwise stated. Images were acquired for 15 min. Every 20 s, a z-stack of 53 GFP images (0.25 µm z-spacing) was acquired, as well as a single, mid-plane differential interference contrast reference image.

For imaging the elevation of the cytoplasmic calcium concentration, 1G4 or non-transduced CD8^+^ T cells were incubated with 2 µM Fura-2 AM (molecular probes) for 30 min at room temperature in imaging buffer. CTL were washed twice thereafter. Because of limited cell numbers, 1G4 SIL were only washed once. T cells were activated with Mel624 APCs as described above, and three images were acquired every 10 s for 15 min: one bright field image, one fluorescence image with excitation at 340 nm, and one fluorescence image with excitation at 380 nm. Imaging data were acquired at 37°C using a 40× oil objective (NA = 1.25) on a Leica DM IRBE-based wide-filed system equipped with Sutter DG5 illumination and a Photometrics Coolsnap HQ2 camera.

Analysis of live cell imaging data using Fiji/ImageJ [37, 38] is described in the Supplementary Materials.

### Flow cytometry

Flow cytometry was executed as previously described [19]. 21-color panel: 1 × 10^6^ cells were first stained with a 1:10 000 dilution of the LIVE/DEAD™ Fixable Red Dead Cell Stain (ThermoFisher) for 15–30 min in the dark at room temperature and washed with FACS buffer (PBS, 0.5% BSA, 2.5 mM EDTA). Fc receptors were blocked with 10 µl of a 1:20 dilution of Fc Blocker (ThermoFisher) in the dark at room temperature for 10 min. 40 µl of biotinylated antibody was added and incubated at 4°C for 30 min. The sample was washed with FACS buffer and incubated with 10 µl of a 1:20 dilution of Monocyte Blocker (BioLegend) in the dark at room temperature for 10 min. 40 µl of an antibody master mix against cell surface proteins as listed in the antibodies section was added for the total volume of 50 µl, incubated at 4°C for 30 min, and washed with FACS buffer. Cells were fixed and permeabilized using the True-Nuclear™ Transcription Factor Buffer Set (BioLegend). Cells were stained for intracellular proteins with an antibody master mix as listed in the antibodies section for 30 min at 4°C. Cells were washed with permeabilization buffer and resuspended in FACS buffer and kept at 4°C until the next day. For flow cytometry data acquisition, samples were run on a four-laser Cytek® Aurora system (4L V/B/YG/R) on low to medium flow rates (15–30 µl/min) and analyzed using the SpectroFlo® software with autofluorescence extraction and live unmix during sample acquisition. The data for the unmixed samples were processed using the FlowJo software (v.10.10.0). Positive gates were set using fluorescence-minus-one data. Cluster analysis of the flow cytometry data is described in the Supplementary Materials.

## Supplementary Material

ltag020_Supplementary_Data

## Data Availability

Raw data are available upon request.

## Permission to reproduce

Elements of figures were created in BioRender: Aljohani, A. (2026) https://BioRender.com/2gdrhva.
