# Supplementary material for: ImmTACs overcome cytotoxic T cell suppression
Source: Immunother Adv. 2026 Aug 14;6(1):ltag020. doi: 10.1093/immadv/ltag020 (PMC13593617; doi:10.1093/immadv/ltag020)
Supplement: Supplementary file 1 [file ltag020_supplementary_data.pdf]

Supplementary Methods

Antibodies

Antibodies and staining reagents used are described in the order: antigen, fluorescent label, clone, supplier, dilution/concentration, RRID:

Panel for flow cytometry

Human CD25, BV421BD, M-A251, BD Bioscience, 1:100, RRID:AB_11154578
Human CD25, BV421BD, 2A3, BD Bioscience, 1:100, RRID:AB_2738555
Human CD8α, Pacific Blue, RPA-T8, BioLegend, 1:25, RRID:AB_493111
Human CD28, BV480, CD28.2, BD Bioscience, 1:100, RRID:AB_2739512
Human CD4, Starbright V570, RPA-T4, Bio-Rad, 1:50, RRID:AB_3099780
Human CD3ε, Super Bright 645, OKT3, eBioscience, 1:20, RRID:AB_2662368
Human CD185 (CXCR5), BV785, J252D4, BioLegend, 1:50, RRID:AB_2629527
Human CD366 (TIM-3), Super Bright 600, F38-2E2, eBioscience, 1:100, RRID:AB_26882087
Human CD45RA, R718, HI100, BD Bioscience, 1:100, RRID:AB_2916420
Human CD279 (PD-1), APC-Cy7, EH12.2H7, BioLegend, 1:100, RRID:AB_10900982
Human CD45, NovaBlue 610-30S, 2D1, eBioscience, 1:100, RRID:AB_3098021
Human CD352 (SLAMF6/Ly108), biotin, REA339, Miltenyi, 1:30, RRID:AB_2657676
Streptavidin, Star Bright Blue 675, Bio-Rad, 1:85
Human CD272 (BTLA), BB700, J168-540, BD Bioscience, 1:50, RRID:AB_2743523
Human CD152 (CTLA-4), PE-Fire 640, BNI3, BioLegend 1:20, RRID:AB_2924561
Human TIGIT, PE-vio770, REA1004, Miltenyi, 1:100, RRID:AB_2751339
Human CD223 (LAG-3), PE-Fire 810, 7H2C65, BioLegend, 1:100, RRID:AB_2927867
Human CD14, RB545, M5E2, BD Bioscience, 1:100, RRID:AB_2691229
Human CD19, RB545, SJ25C1, BD Bioscience, 1:100, RRID:AB_2688586
Human Ki-67, BV711, Ki-67, BioLegend, 1:100, RRID:AB_11218996
Human Foxp3, PE, 259D, BioLegend, 1:50, RRID:AB_492983
Human Foxp3, PE, PCH101, eBioscience, 1:50, RRID:AB_1518782
Human TCF1, Alexa Fluor 647, 7F11A10, BioLegend, 1:50, RRID:AB_2566619
Human T-bet, RB780, O4-46, BD Bioscience, 1:100, RRID:AB_2688938
Live Dead Red, Invitrogen, 1:20,000

Lentiviral transduction and cell culture of human CTL

Blood buffy coats were obtained from healthy donors. PBMC were isolated by density gradient centrifugation using Ficoll-Paque™ (Sigma-Aldrich). PMBC were cryopreserved at a concentration of $3x10^7$ cells/ml. To isolate $CD8^+$ T cells, buffy coat cryopreserved vials were thawed, the cells were washed twice with RPMI 1640 with 10% FBS, 2mM L-glutamine, 50 μM β-mercaptoethanol and resuspended in ice cold MACS buffer. $CD8^+$ T cells were purified by magnetic enrichment for $CD8^+$ cells using CD8 MicroBeads (Miltenyi Biotech). $CD8^+$ T cells were activated using CD3/CD28 Dynabeads (Life Technologies) at bead-to-cell ratio of 1:1 in human IL-2 medium (X-VIVO 15, serum-free hematopoietic cell medium, with 2 mM L-Glutamine and gentamicin (Lonza) supplemented with 5% Human AB serum (Valley Medical), 10 mM neutralized N-acetyl L-Cysteine (Sigma-Aldrich), 50 μM β-Mercaptoethanol (Gibco, ThermoFisher), and 30 U/ml rh-IL-2 (NIH/NCI BRB Preclinical Repository – human IL-2 medium) and incubated overnight at 37°C and 6% CO2.

For the generation of lentiviral particles, HEK 293T cells (RRID:CVCL_0063; Lenti-X 293T cells, Takara) were maintained in DMEM complete medium. $1.5×10^6$ Lenti-X 293T cells were seeded in 5 ml DMEM complete medium in 60x15 mm Primaria culture plates (Corning) 24 h before transfection. Cells were transfected with a total of 4.5 μg plasmid DNA using Fugene HD (Promega): 0.25 μg envelope vector pMD2.G (RRID:Addgene_12259), 2 μg of packaging plasmid pCMV-dR8.91 (Creative Biogene), and 2.25 μg of the pHR_SFFV-based transfer vector. 48 h after transfection, virus containing medium was collected and filtered

through a 0.45 µm nylon filter. For lentiviral infection of CTL, after 24 h of setting up the primary human T cell culture, $1x10^6$ T cells were mixed with 500-700 µl lentivirus-containing medium in a 24-well plate medium in presence of 8 µg/ml Polybrene (Sigma-Aldrich) and centrifuged for 1.5 h at 2500rpm, 32°C. After spinduction primary $CD8^+$ T cells were resuspended in human IL-2 medium. Cells were maintained at densities of less than $2x10^6$ cells/ml and if necessary, spilt back to a density of $0.5\text{-}1x10^6$ cells/ml.

IFNγ secretion

To determine IFNγ secretion, supernatants at the end of the imaging-based cytolysis assays were collected and frozen. The human IFNγ OptEIA ELISA Kits (BD Biosciences) was used according to manufacturer's instructions. Briefly, wells of a 96-well Maxisorb Nunc-Immuno plate (ThermoFisher) were coated with 100 µl anti-human IFNγ monoclonal antibody diluted in 0.1 M Na_2CO_3 coating buffer at a 1:250 dilution and incubated overnight at 4˚C. Plates were blocked using PBS with 10% FBS for 1h. Samples were diluted in ELISA dilution buffer at 1:3 to 1:10 and incubated for 1h at room temperature. 100 µl of working detectors (Biotinylated anti-human IFNγ monoclonal antibody + Streptavidin-horseradish peroxidase conjugate) were incubated for 1 h at room temperature before detection. Absorbance at 450 nm and 570 nm was measured within 20 min. Samples were measured in duplicate or triplicate.

Analysis of live cell imaging data

For analysis of DIC images, tight cell couple formation was defined as the first time point at which a maximally spread immune synapse formed, or 40 s after initial cell contact, whichever occurred first. To assess CTL and SIL morphology in cell couples with tumor target cells, every DIC frame after tight cell couple formation was assessed for the presence of off-synapse lamellae, defined as transient membrane protrusions pointing away from the immune synapse, followed by retraction. To determine CTL translocation over the tumor cell surface, the position of the immune synapse on the tumor target cell was compared to the position at cell coupling. If the T cell had migrated by a distance greater than the diameter of the immune synapse, this was classed as translocation. The formation of a uropod such that it is bound to the tumor cell at the interface rather than pointing away from it together with lamellae at the CTL side opposite of the interface was classed as 'detachment'.

For analysis of F-actin distributions as imaged with F-tractin-GFP, enrichment at the interface and interface center were measured relative to F-actin across the entire cell using Fiji/ImageJ. F-tractin-GFP enrichment across the interface relative to the entire cell was determined in the maximum intensity z-projection of the GFP z-stack. The interface was defined as the 10% of the area of the T cell closest to the T cell/target cell interface. The average fluorescence intensity of the interface area and the entire cell were measured at four to six timepoints after subtracting the mean of ten background fluorescence readings. F-tractin-GFP enrichment at the centre of the interface relative to the entire cell was determined in the midplane of the GFP z-stack of the T cell of interest. The area of the interface centre was defined as the middle third of the interface as defined above, that is as 3.5% of the total cell midplane area. The average fluorescence intensity of the interface center and the entire cell were measured at four to six timepoints after subtracting the mean of ten background fluorescence readings.

For analysis of the Fura-2 imaging experiments using Fiji/ImageJ, rolling ball background fluorescence was subtracted from the fluorescence data and the ratio of the Fura-2 images upon excitation at 340 nm versus 380 nm was calculated and multiplied by 100 to fit into the 8-bit display scale. Average ratio within a circular region of interest of the dimensions of the T cell was determined over time for each T cell.

Cluster analysis of flow cytometry data

For cluster analysis, samples were gated for live, singlet, $CD45^+CD3^+CD19^-CD14^-CD8^+$ cells. The FlowJo 10.10.0 clean plugin was used to reduce technical noise. Files for experimental repeats were concatenated with the FlowJo concatenation tool. $CD8^+$ cells for each experimental repeat were down sampled to 20,000 cell and analyzed for CD25, CD28, CD185, CD366, CD45RA, CD279, CD352, CD272, CD152, TIGIT, CD223, Ki-67, FOXP3, TCF1, T-BET. t-SNE (t-Distribution Stochastic Neighbor Embedding) was employed to map the high-dimensional cellular data into a two-dimensional space using: iteration: 1000, perplexity: 30, K-nearest neighbors' algorithm: Exact (vantage point tree), and gradient algorithm: Barnes-Hut. Cells were clustered by implementing FlowSOM v. 3.0.18 with 10 metaclusters and 10 × 10 grid size, all other parameters were left at the default settings.

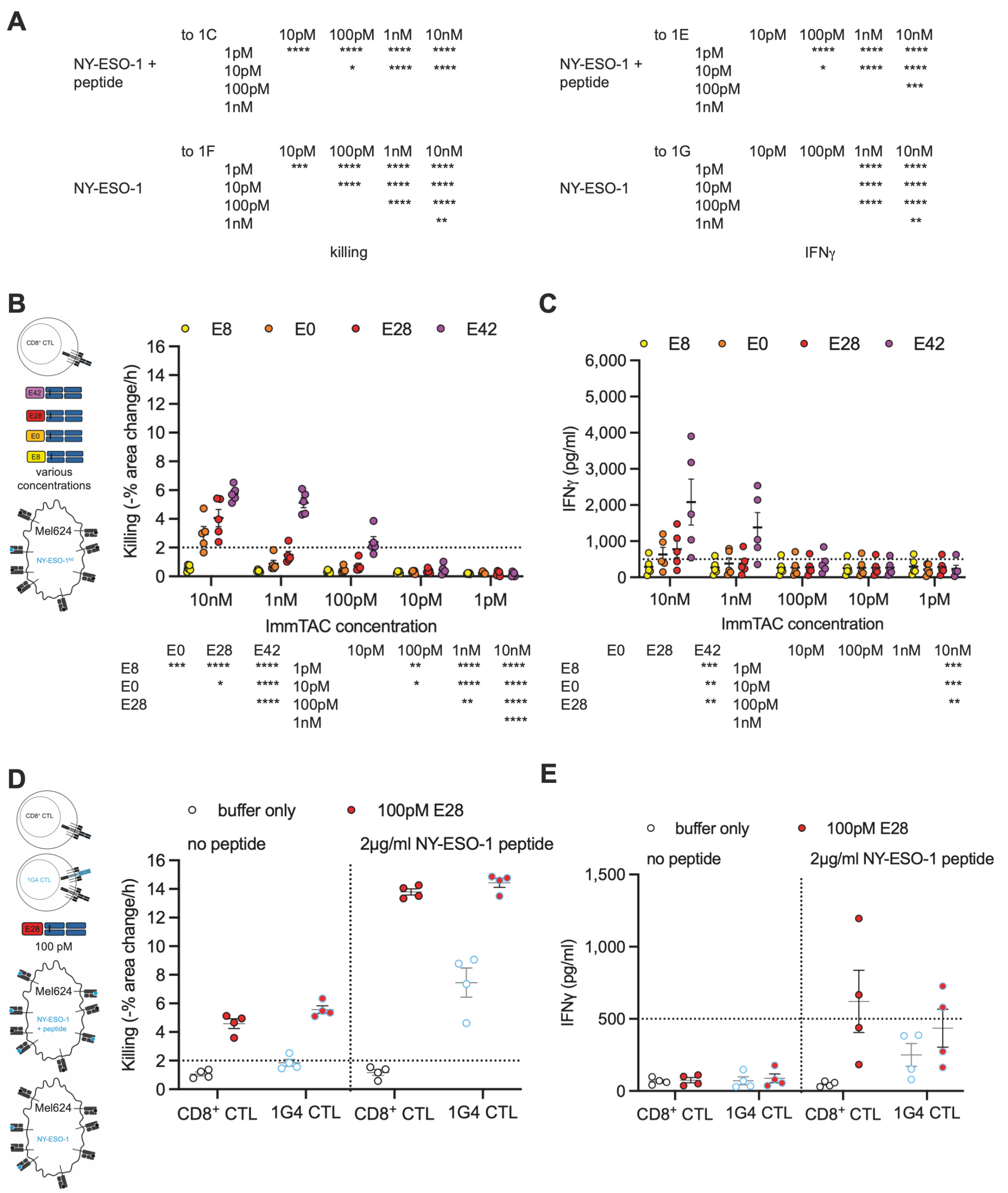

Fig. S1 ImmTAC-dependent CTL effector function is reduced upon knockdown of NY-ESO-1

A, B Killing (A) of Mel624 NY-ESO-1 knockdown tumor target cells by non-transduced CTL in the presence of the indicated concentration of the given ImmTAC version and (B) IFNγ amounts in corresponding supernatants; mean ± SEM. 5 independent experiments. Statistical significance determined by paired Two-way ANOVA and given as main effects of ImmTAC version and concentration in the two tables at the right. **C, D** Killing (C) of Mel624 tumor target cells in the presence of exogenous NY-ESO-1 agonist peptide or not by non-transduced CD8⁺ (black circle) or 1G4 CTL (blue circle) in the presence or absence of 100 pM of the E28 ImmTAC and (D) IFNγ amounts in corresponding supernatants; mean ± SEM. 4 independent experiments.

A

to 3C

	6	8	10	12	14
4				*	***
6				*	***
8					**
10					*
12					

to 3E for E42

	6	8	10	12	14
4				**	****
6					***
8					**
10					
12					

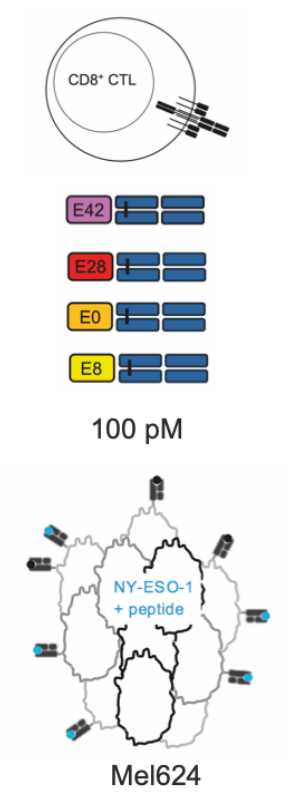

B

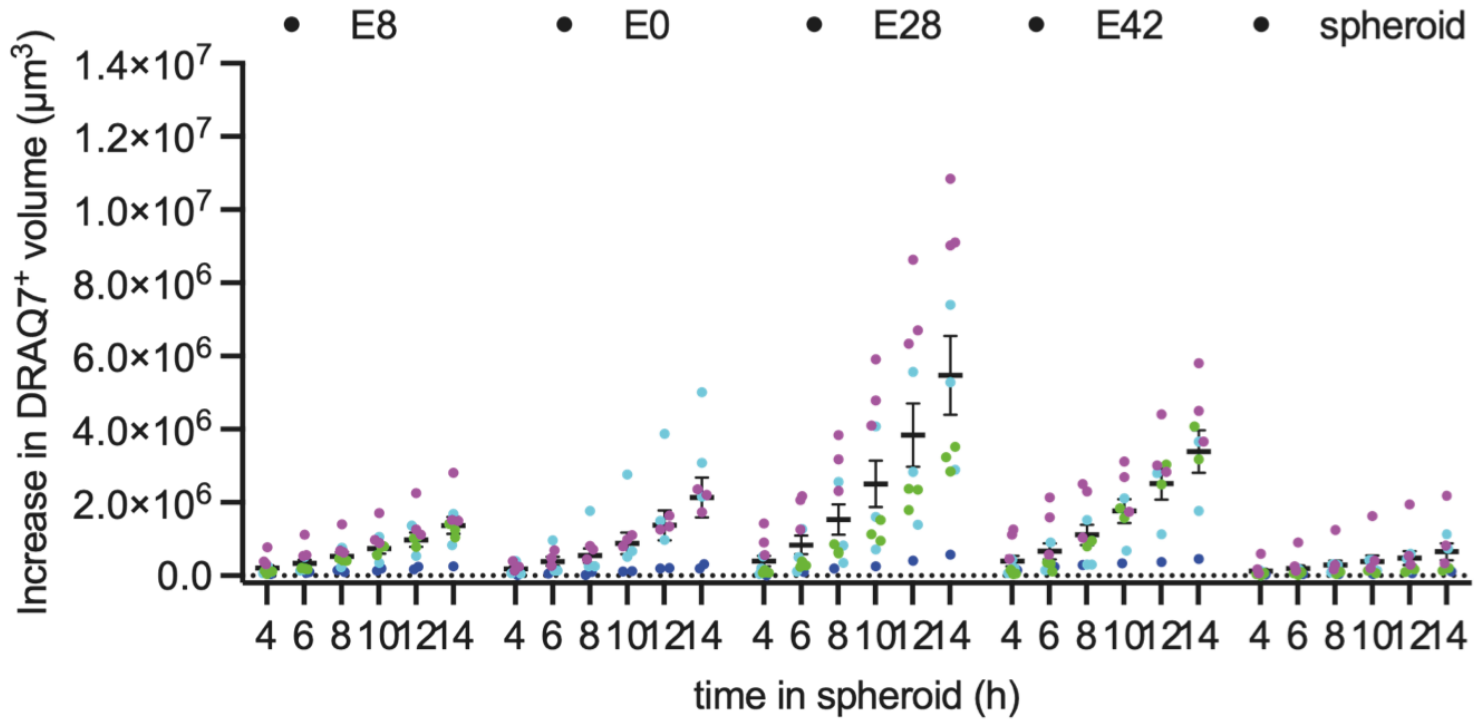

C

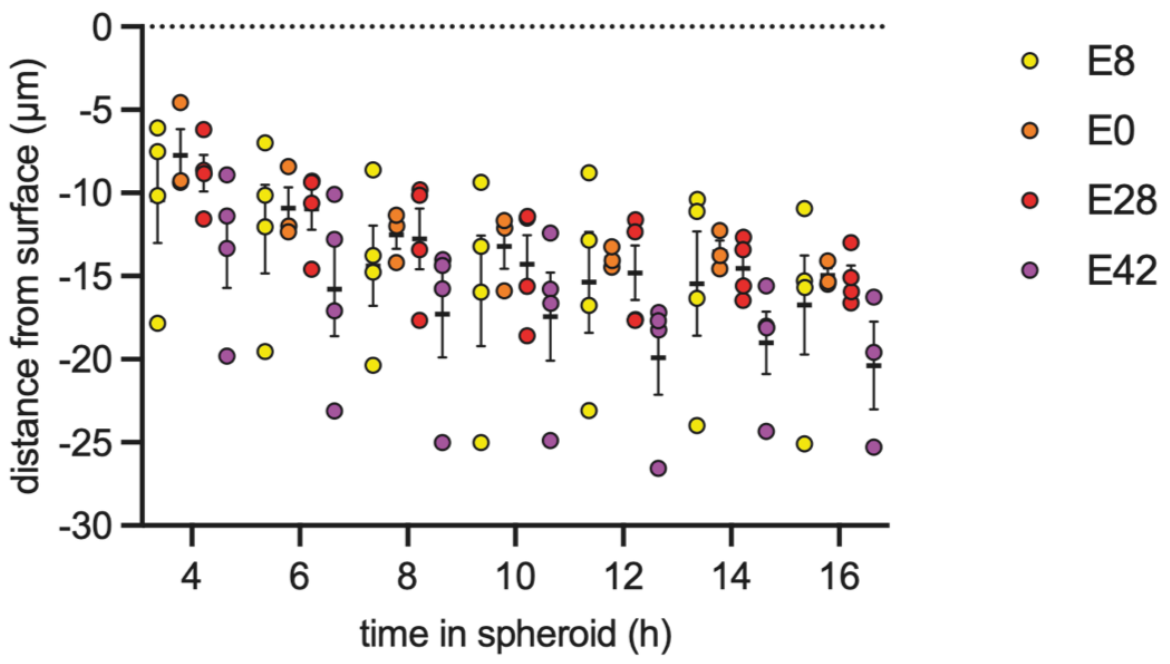

	6	8	10	12	14	16
4	*	***	****	****	****	****
6			*	**	**	**
8						*
10						
12						

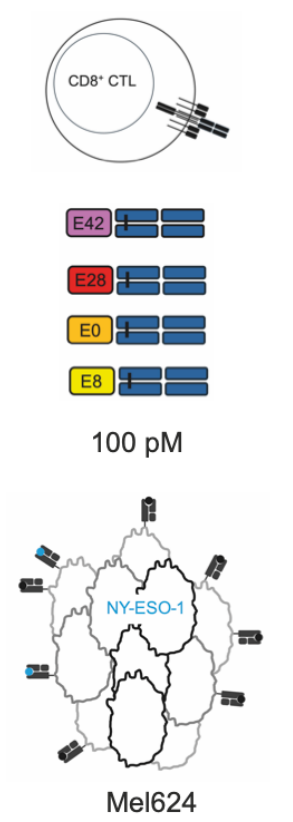

D

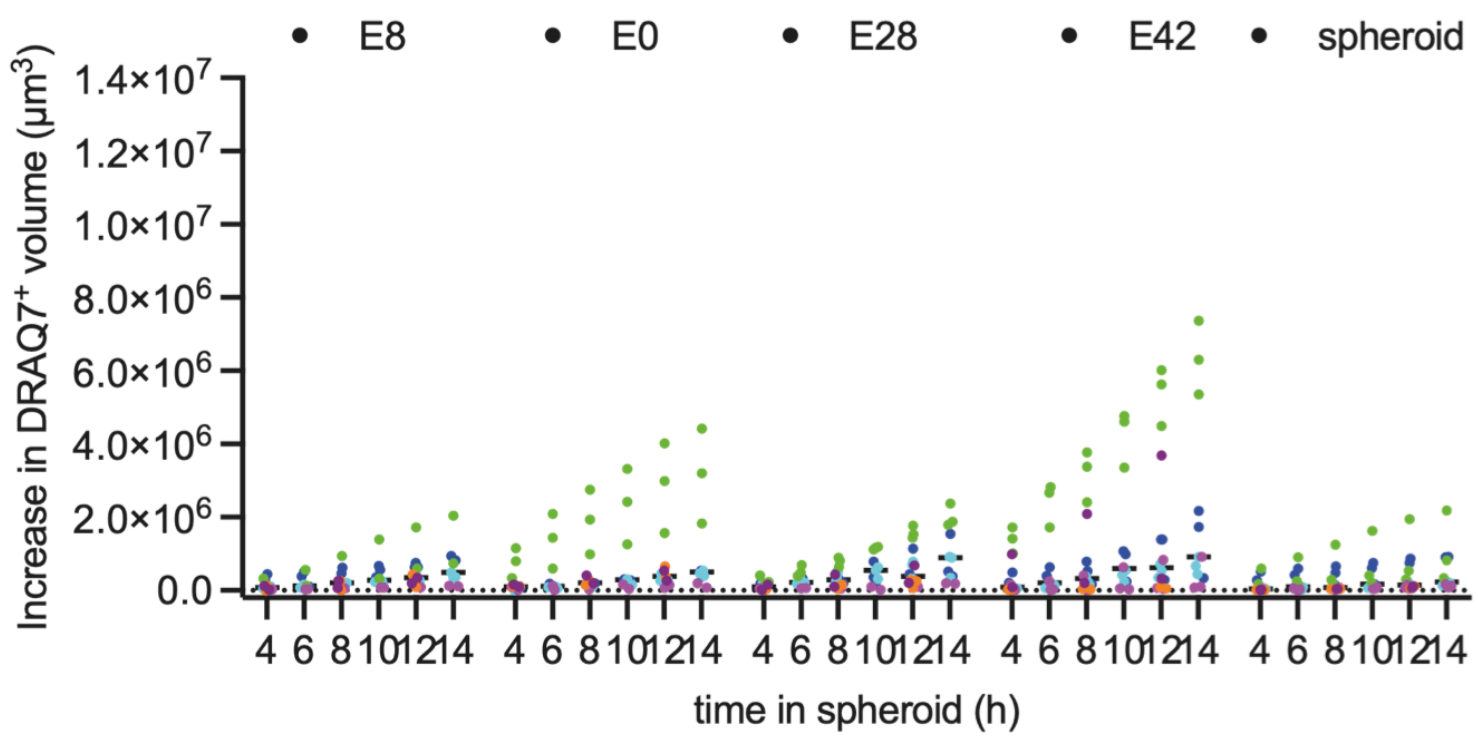

E

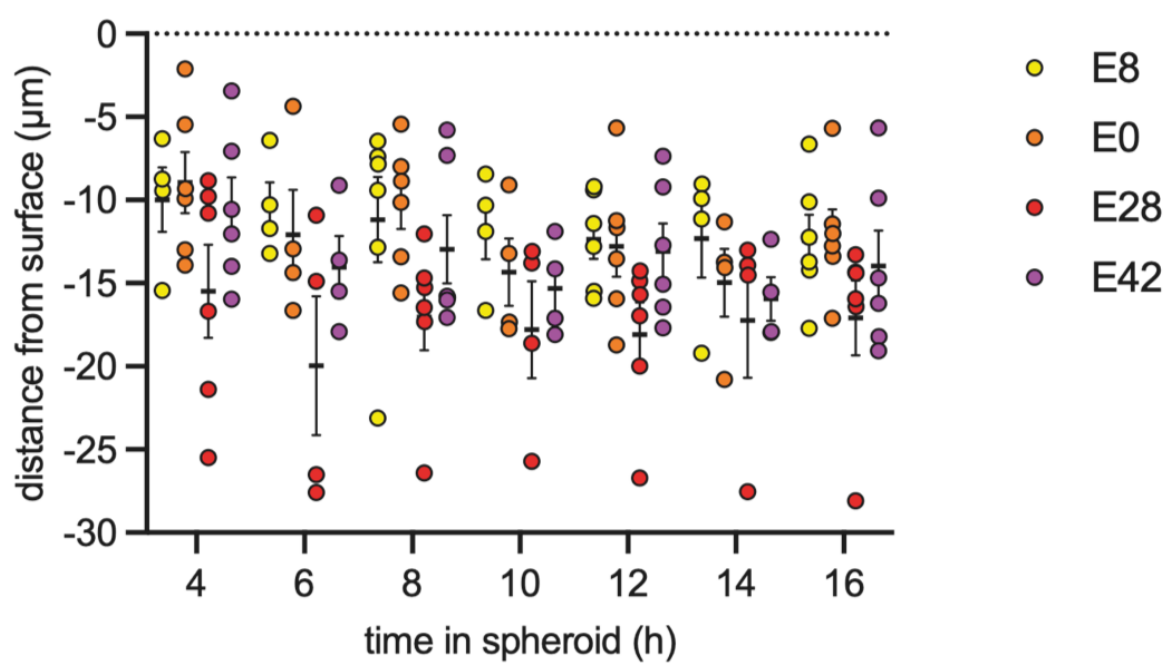

	E0	E28	E42
E8		*	
E0		*	
E28			

	6	8	10	12	14	16
4				*		*
6						
8						
10						
12						

Fig. S2 ImmTACs mediate target cell killing in tumor cell spheroids

A Single spheroid data for Fig. 3C. ImmTACs are not identified by color but are given from left to right in the order of E8 to E42 as indicated above the data points. 'Spheroid' is the no

CTL control. Spheroids from the same experiment share their color across all experimental conditions. **B** SIL infiltration depths for the same experiments as in Fig. 3C; mean ± SEM. Statistical significance determined by paired Mixed-effects analysis and given as main effect of time in the table at the right. **C** Single spheroid data for Fig. 3E as in A. **D** SIL infiltration depths for the same experiments as in Fig. 3E; mean ± SEM. Statistical significance determined by paired Mixed-effects analysis and given as main effects of ImmTAC version and time in the tables at the right.

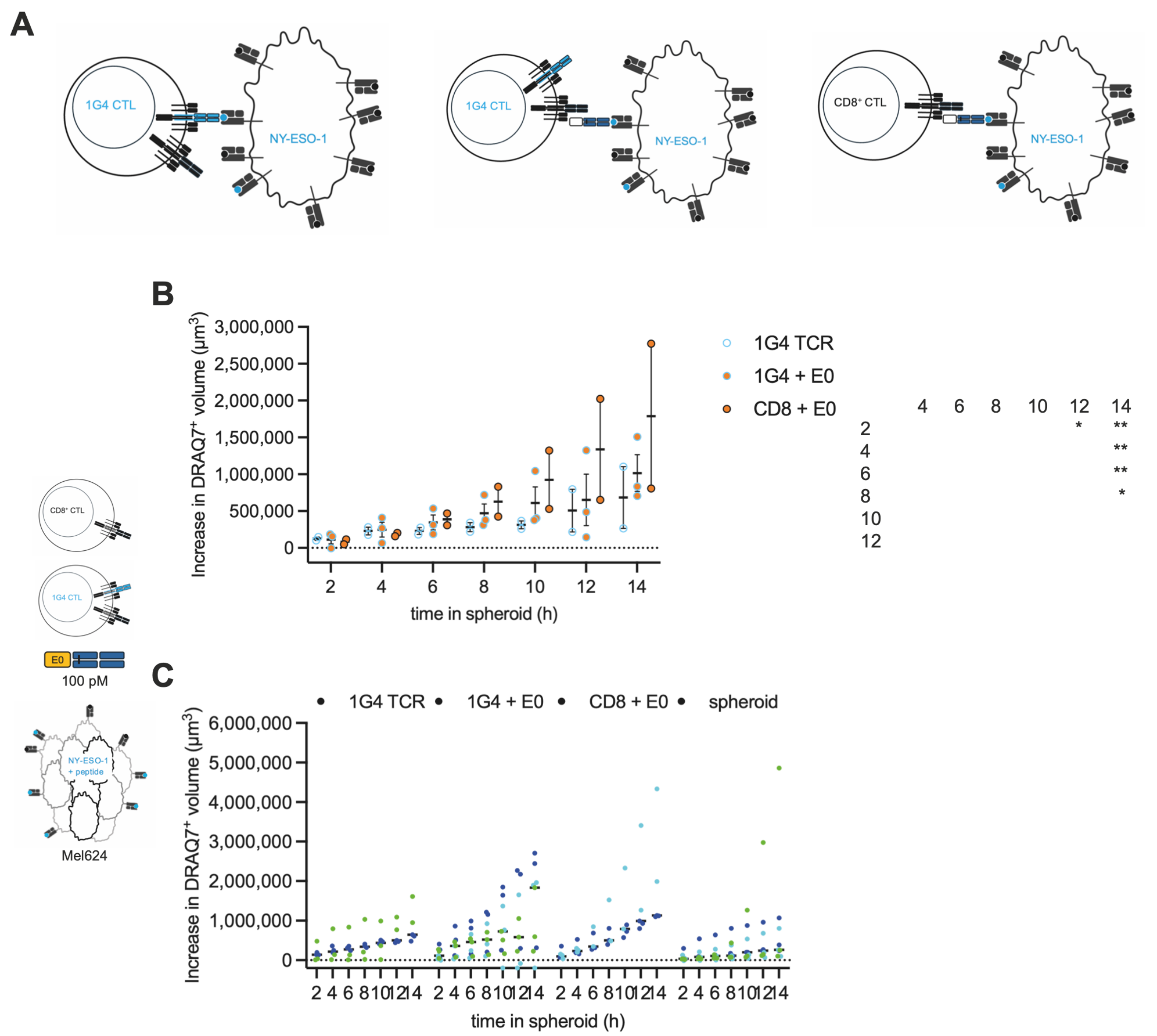

Fig. S3 Comparable target cell killing efficiency of tumor cell spheroids elicited by CTL activated via peptide-MHC or ImmTACs

A Graphical depiction of the direct activation of 1G4 CTL by NY-ESO-$1_{157\text{-}165}$/HLA-A*02:01 versus the indirect activation of 1G4 or non-transduced CTL via ImmTACs recognizing the same NY-ESO-$1_{157\text{-}165}$/HLA-A*02:01 complex. **B** 1G4 and non-transduced CTL cocultured with Mel624 spheroids incubated with 2 µg/ml NY-ESO-1 peptide with and without 100 pM E0 ImmTAC. Each data point is an independent experiment (N=2, 3) with a total of 6-9 spheroids analyzed per condition. Statistical significance determined by paired Mixed-effects analysis and given as main effect of time in the table at the right. Single spheroid data are given in C. **C** Experimental conditions are not identified by color but are given from left to right in the order of 1G4 CTL, 1G4 CTL plus 100 pM E0 and $CD8^{+}$ CTL plus 100 pM E0, as indicated above the data points. 'Spheroid' is the no CTL control. Spheroids from the same experiment share their color across all experimental conditions.. * $p<0.05$, ** $p<0.01$.

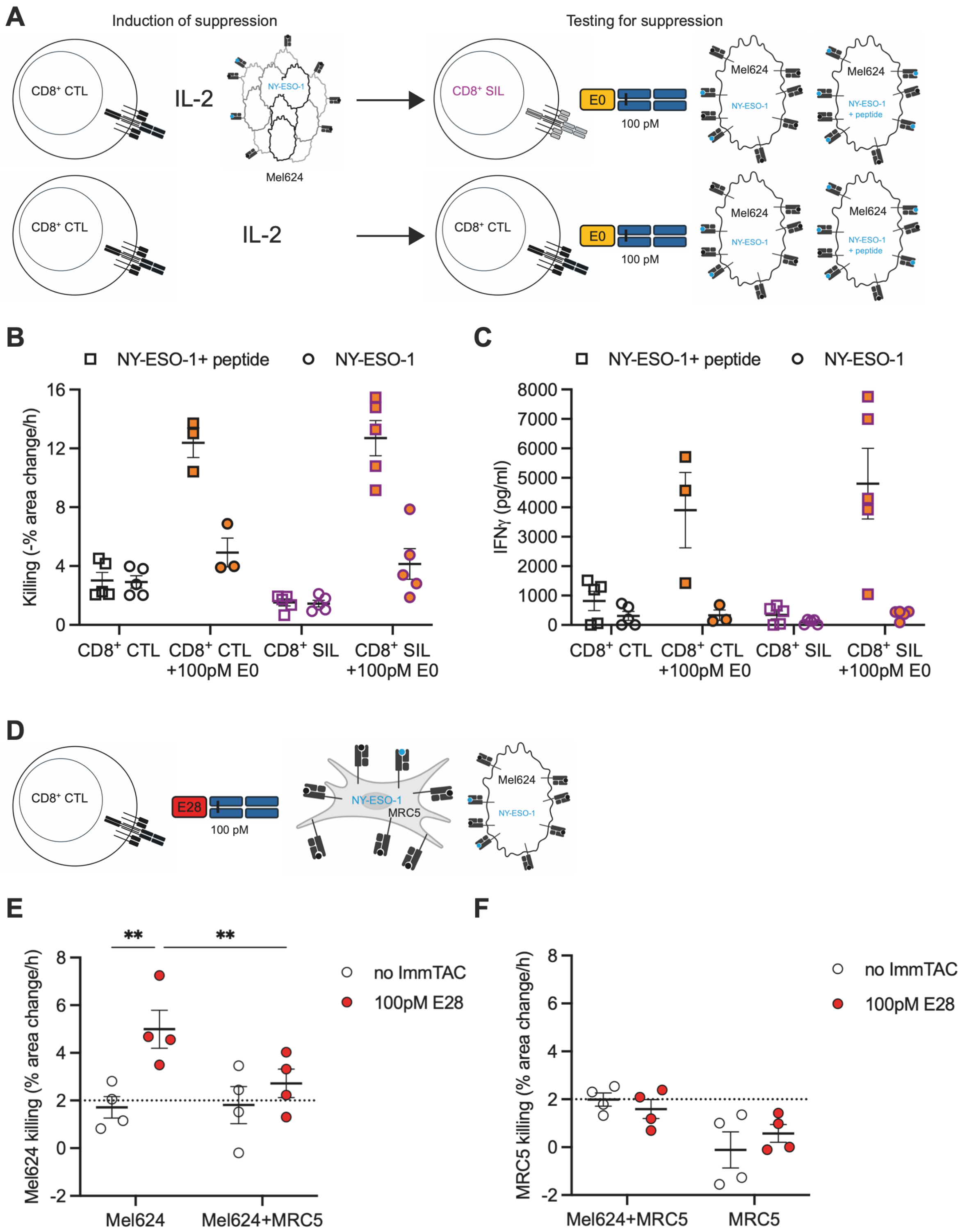

Fig. S4 TCR engagement with antigen is required for the induction of CTL suppression in spheroids

A Graphical depiction of the experiment to test induction of suppression by CTL incubation in spheroids in the absence of antigen and the subsequent experiment to test for such suppression (top row) and of the corresponding non-suppressed control experiment (bottom row). **B, C** Killing (B) of Mel624 tumor target cells with or without incubation with 2 µg/ml NY-ESO-1 peptide by non-transduced CTL previously incubated or not in spheroids without antigen exposure in the absence or presence of 100 pM E0 ImmTAC and (C) IFNγ amounts

in the corresponding supernatants; mean ± SEM. 3-5 independent experiments. **D** Graphical depiction of the experiment to test suppression of CTL function in response to ImmTAC by CAFs. **E, F** Killing of Mel624 tumor target cells (E) or MRC5 CAF (F) with or without incubation with 100 pM E28 by non-transduced CTL. A negative killing rate indicates faster cell proliferation than control; mean ± SEM. 4 independent experiments. Statistical significance determined by paired Two-way ANOVA. ** $p<0.01$.

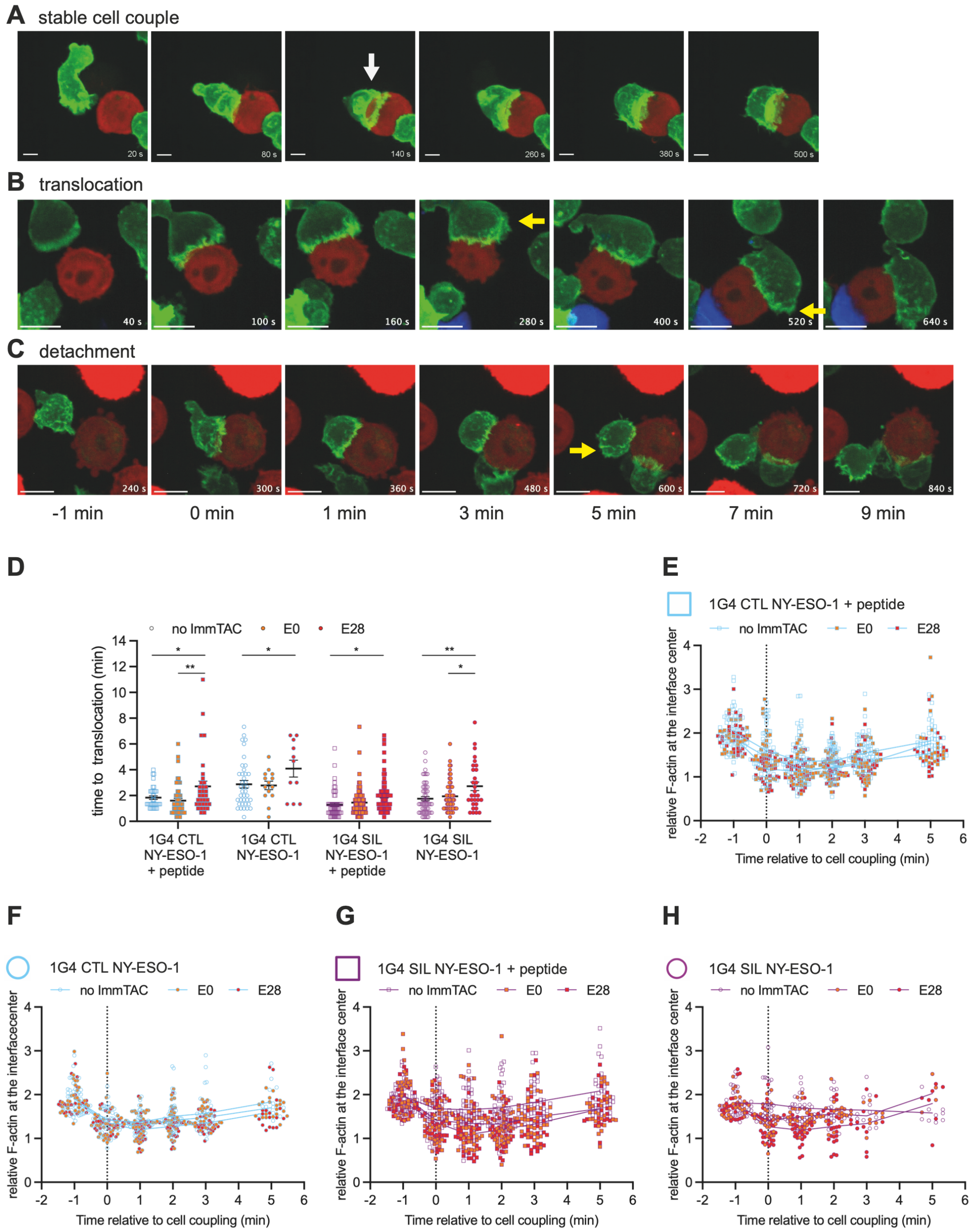

Fig. S5 ImmTACs effectively induce cytoskeletal polarization of CTL interacting with tumor target cells

A-C Maximum projections of 3D imaging data for the representative phenotypes as indicated of the interaction of 1G4 CTL transduced to express F-tractin-GFP (green) and $CD8^+$ CTL (blue) with Mel624 target cells (red) at the indicated times relative to the formation of a tight cell couple. The white arrow denotes F-actin clearance at the interface center, the yellow arrow off interface lamellae. Scale bar=5µm. **D** Time to translocation in the interaction of 1G4 CTL or SIL with Mel624 cells in the presence or absence of 2 µg/ml NY-ESO-1 agonist peptide and 100 pM of the indicated ImmTAC; mean ± SEM. Part of the same experiments as Fig. 5 A-D. Statistical significance determined by One-way ANOVA. **E-H** Single cell data for Fig. 5E-H. On average 36 (26-64) cell couples analyzed per condition. * p<0.05, ** p<0.01.

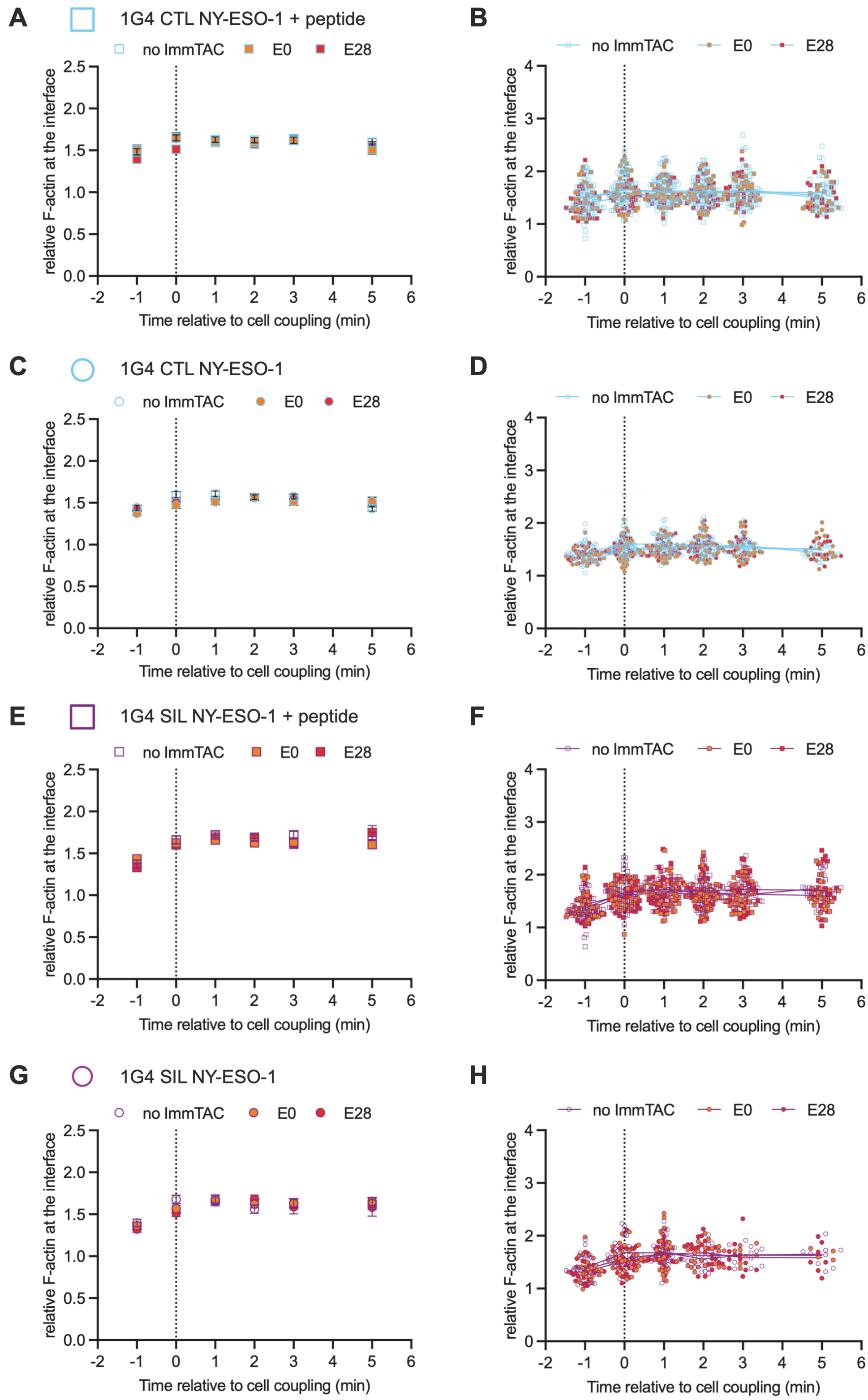

Fig. S6 ImmTACs effectively induce cytoskeletal polarization of CTL interacting with tumor target cells

A-H F-actin accumulation at the entire interface between 1G4 CTL (A-D) and 1G4 SIL (E-H) expressing F-tractin-GFP and Mel624 cells in the presence (A, B, E, F) or absence (C, D, G, H) of NY-ESO-1 agonist peptide and 100 pM of the indicated ImmTAC relative to F-actin in the entire cell and to the time of tight cell coupling. Pooled data from 2-3 independent experiments. A, C, E, G Experiment averages; mean ± SEM. B, D, F, H Corresponding single cell data. On average 36 (26-64) cell couples analyzed per condition.

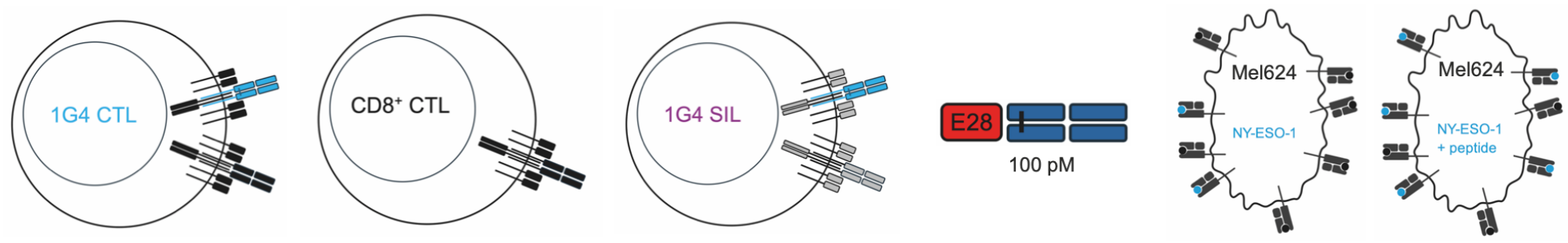

A

	1G4 CTL	1G4 CTL peptide	1G4 CTL E28
1G4 CTL, peptide	****		
1G4 CTL, E28		****	
1G4 CTL, E28, peptide	****	****	

	CD8 CTL	CD8 CTL peptide	CD8 CTL E28
CD8 CTL, peptide			
CD8 CTL, E28			
CD8 CTL, E28, peptide	****	****	****

	1G4 SIL	1G4 SIL peptide	1G4 SIL E28
1G4 SIL, peptide			
1G4 SIL, E28	***	*	
1G4 SIL, E28, peptide	****	****	****

B

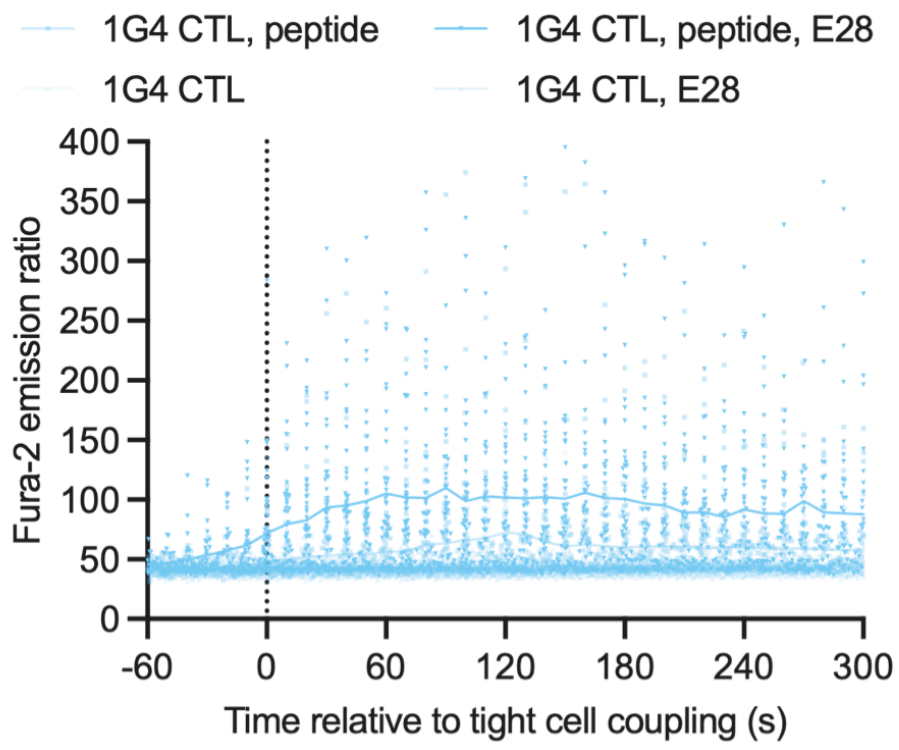

	1G4 CTL	1G4 CTL peptide	1G4 CTL E28
1G4 CTL, peptide	****		
1G4 CTL, E28		****	
1G4 CTL, E28, peptide	****	****	****

C

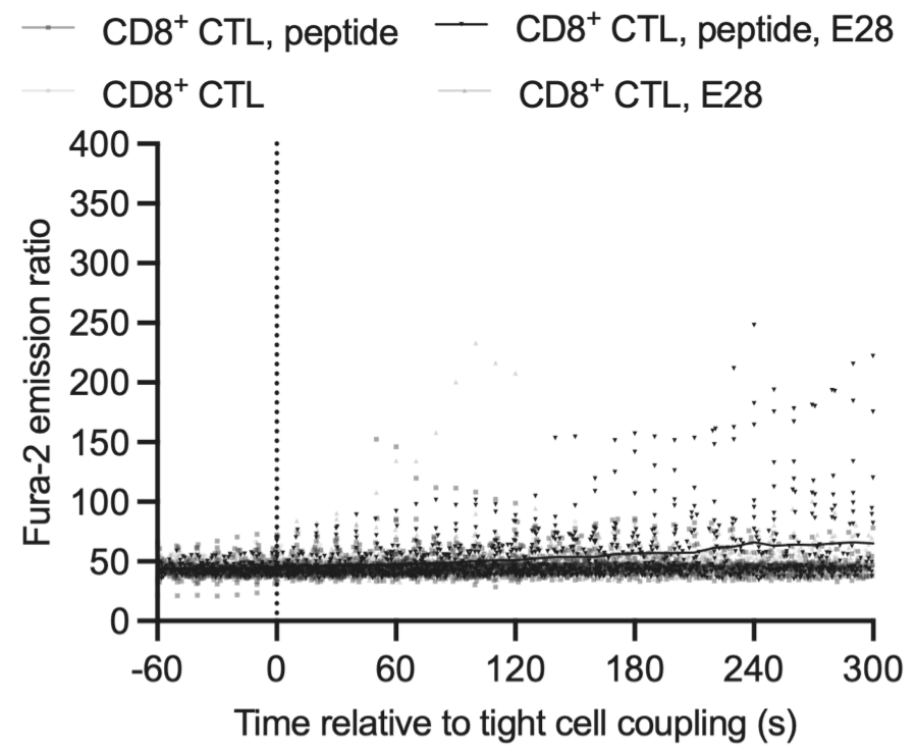

	CD8 CTL	CD8 CTL peptide	CD8 CTL E28
CD8 CTL, peptide			
CD8 CTL, E28		*	
CD8 CTL, E28, peptide	****	****	****

D

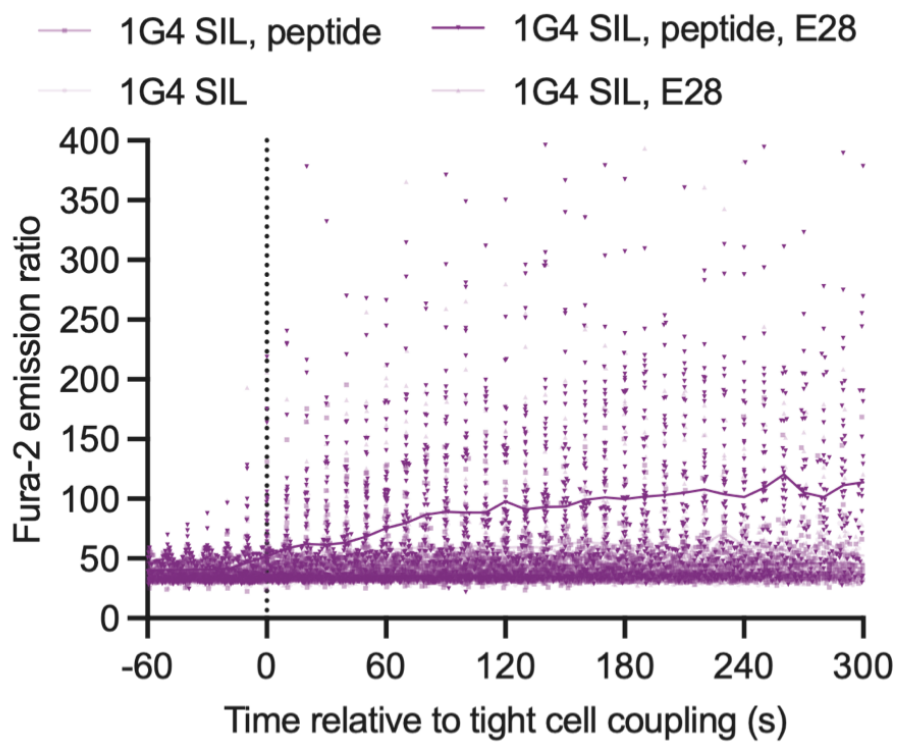

	1G4 SIL	1G4 SIL peptide	1G4 SIL E28
1G4 SIL, peptide			
1G4 SIL, E28	****	****	
1G4 SIL, E28, peptide	****	****	****

Fig. S7 E28 ImmTAC triggers the elevation of the CTL cytoplasmic calcium concentration more effectively than direct engagement of a TCR by peptide-MHC
A Statistical significance for the data in Fig. 6 B-D. **B-D** Single cell data for Fig. 6B-D, respectively, as pooled over all independent experiments of a given condition. 32-117 cells analyzed per condition. Statistical significance determined by paired Mixed-effects analysis and given in the table at the bottom. * p<0.05, *** p<0.001, **** p<0.0001.

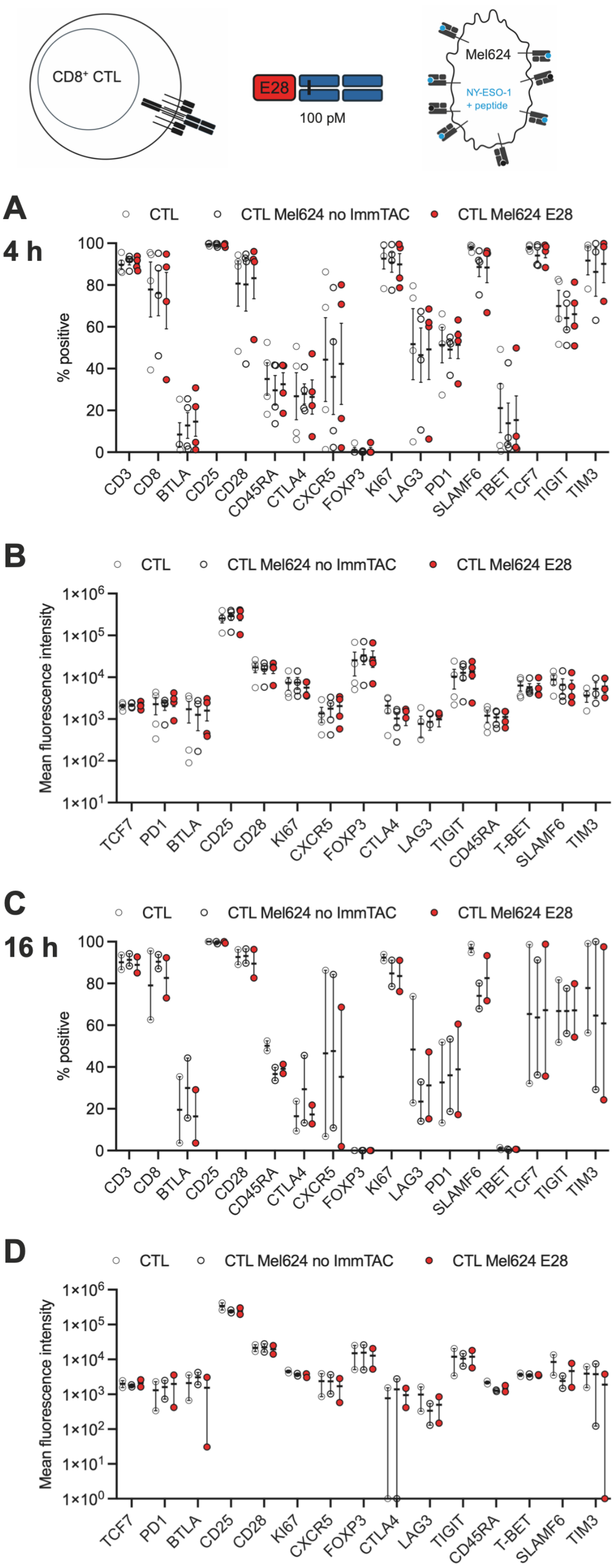

Fig. S8 E28 ImmTAC does not trigger substantial changes in T cell marker expression on the time scale of the execution of CTL effector function

Percentage of $CD8^+$ CTL positive for the indicated marker (A, C) and mean fluorescence intensity (B, D) in $CD8^+$ CTL, $CD8^+$ CTL after 4 h (A, B) and 16 h (C, D) of incubation with Mel624 cells in the presence of exogenous NY-ESO-1 agonist peptide with or without 100 pm E28. 4 independent experiments. Representative flow cytometry data in Fig. S9.

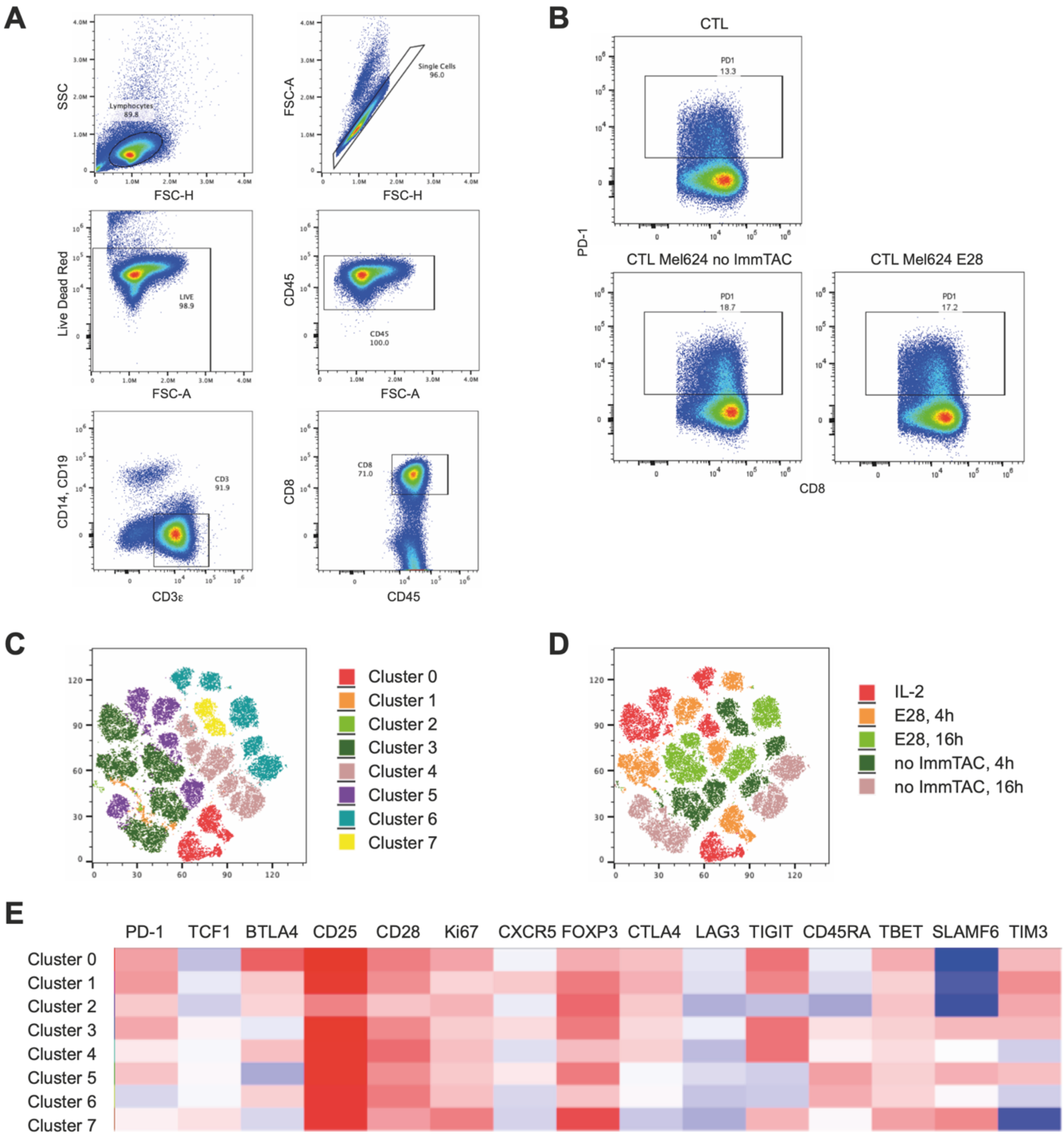

Fig. S9 E28 ImmTAC does not trigger substantial changes in T cell marker expression on the time scale of the execution of CTL effector function
A Gating strategy **B** Representative flow cytometry data. **C-E** Cluster analysis of the same data as in Fig. S8 as, (C) a t-SNE blot of clusters identified, (D) overlay of the five experimental conditions and (E) expression of the indicated markers.
